# Oxysulfonylation of Alkynes with Sodium Sulfinates to Access β-Keto Sulfones Catalyzed by BF_3_·OEt_2_

**DOI:** 10.3390/molecules29153559

**Published:** 2024-07-28

**Authors:** Shi-Wei Yu, Zu-Jia Chen, Huan-Qing Li, Wen-Xi Li, Yun Li, Zong Li, Zhao-Yang Wang

**Affiliations:** School of Chemistry, South China Normal University, Guangzhou Key Laboratory of Analytical Chemistry for Biomedicine, GDMPA Key Laboratory for Process Control and Quality Evaluation of Chiral Pharmaceuticals, Key Laboratory of Theoretical Chemistry of Environment, Ministry of Education, Guangzhou 510006, China; 2021022671@m.scnu.edu.cn (S.-W.Y.); 2022022607@m.scnu.edu.cn (Z.-J.C.); 2022022611@m.scnu.edu.cn (H.-Q.L.); 2023022646@m.scnu.edu.cn (W.-X.L.); m15192479187@163.com (Y.L.); lzscnu@outlook.com (Z.L.)

**Keywords:** sodium sulfinates, alkynes, sulfonylation reaction, BF_3_·OEt_2_, β-Keto sulfones

## Abstract

An efficient and operationally simple method for the synthesis of β-keto sulfones through the BF_3_·OEt_2_-promoted reaction of alkynes and sodium sulfinates is developed. With its facile and selective access to the targets, it features good functional group compatibility, mild conditions, easily available starting materials, and good yields. Notably, the reaction does not require metal catalysts or chemical reagents with pungent odors.

## 1. Introduction

**Figure 1:**
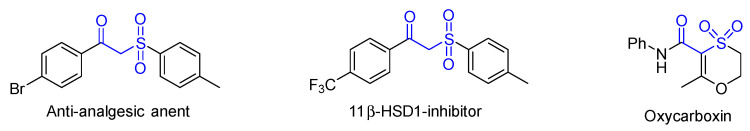
Some bioactive β-keto sulfone derivatives.

Sulfone compounds are of considerable importance in synthetic and medicinal chemistry [1,2]. For example, the introduction of sulfonyl groups into medicines can substantially influence their polarity, acidity, aqueous solubility, and other properties [3,4], so the efficient synthesis of sulfone compounds has attracted extensive attention recently [5,6]. Among them, β-keto sulfone as a kind of unique sulfone compound has been extensively used in biomedicine, such as for anti-schistosomal, anti-analgesic, and antibacterial effects (Figure 1) [7,8,9]. At the same time, it can also be used as a synthon and is widely used in synthetic chemistry [10,11,12].

Traditionally, β-keto sulfones can be synthesized through the nucleophilic alkylation of sodium sulfinates with acyl halides [13]. In recent years, with the rise of green synthetic chemistry and the development of various sulfur reagents [14,15,16,17], the coupling reaction of unsaturated bonds (e.g., alkynes) and sulfur reagents has gradually become the main way to synthesize β-keto sulfones [18,19,20]. Among them, being bench-stable, commercially or readily available, and easy to handle [21,22], sodium sulfinates/sulfinic acid is widely used in the synthesis of β-keto sulfones [23,24,25,26].

**Scheme 1:**
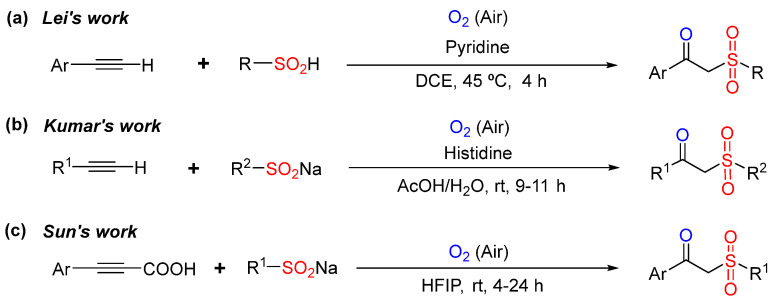
Examples of access to β-keto sulfones starting from sodium sulfinates or sulfinic acids [27,28,29].

For instance, Lei’s group [27] reported the oxysulfonylation of terminal alkynes with sulfinic acids catalyzed by pyridine (Scheme 1a). Afterward, Kumar’s group [28] also reported a similar reaction (Scheme 1b). Recently, Sun’s group [29] reported the oxysulfonylation of arylpropiolic acids and sodium sulfnates to generate β-keto sulfones using only hexafluoroisopropanol (HFIP) as a solvent and oxygen as a green oxidant (Scheme 1c).

**Scheme 2:**
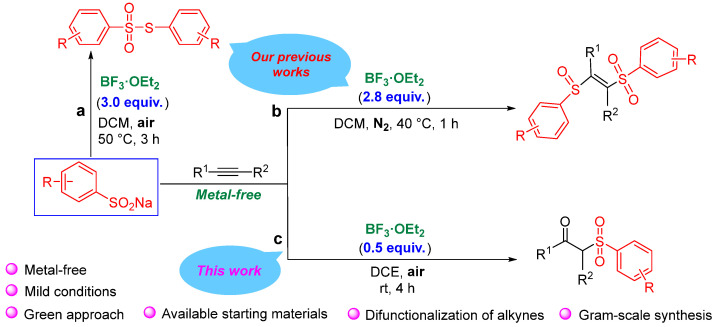
Serial synthesis reactions of sodium sulfinates catalyzed by BF_3_·OEt_2_ [34,35].

On the other hand, BF_3_·OEt_2_ exhibits strong Lewis acidity [30,31], and its excellent catalytic activity is widely used in various organic synthesis reactions [32,33]. In our previous work [34], we reported a BF_3_·OEt_2_-mediated difunctionalization reaction of sodium sulfinates and alkynes to obtain β-sulfinyl alkenylsulfone (Scheme 2b). In fact, during the condition optimization for the above-mentioned work, we also unexpectedly found that if the amount of BF_3_·OEt_2_ was below 1.0 equiv., the main product changed from β-sulfinyl alkenylsulfones to β-keto sulfones.

In view of this, based on our previous research on C-S bond construction [21,36], especially the sodium sulfinate reaction with BF_3_·OEt_2_ as a catalyst [34,35], herein, we hope to report a new reaction under air atmosphere to efficiently obtain β-keto sulfones via the radical pathway (Scheme 2c). This reaction is designed to synthesize β-keto sulfones without any metal catalysts and does not require the use of chemical reagents with irritating odors such as pyridine and acetic acid. Therefore, this method is more gentle and greener and its successful development is also conducive to expanding the application of BF_3_·OEt_2_ as a catalyst. In particular, the regulation and control through this organosulfur reagent and BF_3_·OEt_2_ catalyst under different reaction conditions can synthesize different products, such as thiosulfonates, β-sulfinyl alkenylsulfones, and β-keto sulfones. Thus, this strategy is of great significance in the fields of organic sulfur chemistry, BF_3_ chemistry, and alkyne conversion.

## 2. Results and Discussion

### 2.1. Optimization of Reaction Conditions

We attempted the reaction of phenylacetylene (**1a**) and sodium *p*-tolylsulfinate (**2a**) as a model in dichloromethane (DCM) to systematically investigate the influences of different factors to optimize the best conditions. The results are summarized in Table 1.

Initially, we examined the reaction time (Entries 1–3). When the reaction time increased to 6 h, there was no improvement. When the reaction time was shortened, the yield decreased sharply.

In addition, lowering or increasing the reaction temperature did not increase the yield (Entries 4–5 vs. Entry 1).

Similarly, we also examined the amount of BF_3_·OEt_2_ used. It was found that increasing or decreasing the amount of Lewis acid did not further increase the yield (Entries 6–7 vs. Entry 1).

At the same time, the effect of solvents on the model reaction was investigated (Entries 1, 8–22). It was found that the solvent plays a significant role in the success of this reaction. Among different solvents, e.g., ethyl acetate (EA), dimethyl sulfoxide (DMSO), tetrahydrofuran (THF), and *N*,*N*-dimethylformamide (DMF), using 1,2-dichloroethane (DCE) as the solvent was more favorable to the formation of **3a** (Entry 8 vs. Entry 1). Therefore, we decided to choose DCE as the best solvent.

Furthermore, we investigated the ratio of reactants **1a** and **2a**. When the ratio of **1a**:**2a** was 1:2.4, the best effect was achieved (Entry 8 vs. Entries 23–24).

Thus, the optimized reaction conditions were identified as using **1a** (0.3 mmol), **2a** (0.72 mmol), and BF_3_·OEt_2_ (0.15 mmol) as the catalyst and 4.0 mL of DCE as the solvent at room temperature (rt) for 4 h.

### 2.2. Scope of Substrates

Under the optimized conditions, a range of alkynes **1** and sodium sulfinates **2** were applied in the transformation to establish the scope and generality of this reaction.

We first investigated various alkyne substrates **1** to explore the scope of this reaction (Table 2).

Pleasingly, many arylalkynes **1** bearing electron-donating substituents (e.g., methyl, ethyl, and *tert*-butyl) at the para-position of the aryl group were successfully employed with moderate yields (**3a**-**3d**, 50–63%). For other substituents, e.g., halogen, there was a slight decrease in the yield of the desired product (**3e**-**3g**, 38–51%). In particular, when the strong electron-withdrawing groups (e.g., -CF_3_, -NO_2_) were bearing in **1**, the yield decreased further (**3h**-**3i**, 32–35%).

In the next step, the effect of different positions of the arylalkyne ring on the yield was investigated. Due to the steric effect, for the same substituent group, when it was a *meta*-substituted group in the arylalkyne substrate **1**, the yield was lower than that of the *para*-substituted group, such as **3b** (58%) vs. **3j** (50%), **3f** (48%) vs. **3l** (42%), and **3g** (51%) vs. **3m** (47%).

As anticipated, when the arylalkyne substrate **1** was bearing *ortho*-substituted, the yield was further reduced, such as **3b** (58%) vs. **3j** (50%) vs. **3n** (40%), and **3k** (55%) vs. **3o** (49%). Furthermore, polycyclic substituted alkynes **1** also can be transformed into the corresponding products (e.g., **3p**, 46%).

Importantly, the scope of sodium arylsulfinate substrates **2** was also examined. The results are also summarized in Table 2. Obviously, the substituted **2** was able to react with **1a** to produce the corresponding products with moderate yields (**3q-3t**, 51–67%). Among them, the substrate **2** containing electron-donating groups performed better than those with electron-withdrawing groups, such as **3r** (67%) vs. **3a** (56%) vs. **3q** (51%) vs. **3s** (50%), and **3c** (63%) vs. **3t** (62%).

### 2.3. Structural Characterization Analysis

From the ^1^H NMR spectra of the target compounds, it can be seen that the ^1^H NMR data of the twenty compounds **3a**-**3t** are consistent with the simulated data of the hydrogen atom in the target products. For example, the ^1^H NMR spectra of the synthesized compounds **3a**-**3t** show that there was a single peak with the integration of two units near 4.72 ppm, which was a characteristic peak caused by methylene hydrogen in the structure of the target products.

At the same time, in the ^13^C NMR spectra, the single peak of the chemical shift value near 64.0 ppm was also a characteristic peak of methylene carbon. In addition, regardless of whether the product contains one fluorine atom or trifluoromethyl group, the corresponding chemical shift can be found in ^19^F NMR, as anticipated.

In a word, these test results indicate that the characterization results of NMR are consistent with expectations. The tested data of the synthesized compounds **3a**-**3t**, including the data of the melting point (m.p.), are also consistent with data in the references reported before [17,19,23,25].

Although NMR tests have proven that compounds **3a**-**3t** have the expected structure, in order to further determine the structure of the product, we also conducted single-crystal cultivation of compound **3a**. Its crystal resolution data are shown in Table 3, successfully affirming that **3a** does have the expected structure [37].

**Figure 2:**
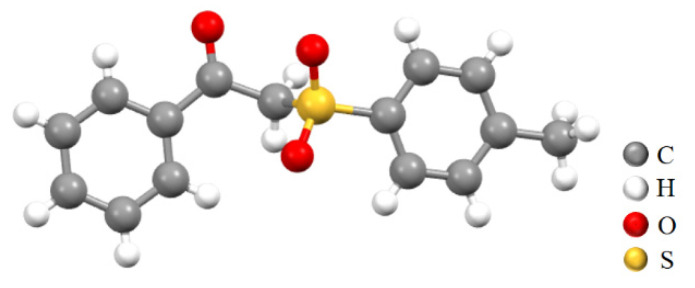
The crystal structure of **3a** (**CCDC**: 2367445).

Accordingly, the X-ray single-crystal diffraction results of compound **3a** in Figure 2 indicate that the molecule contains two benzene rings, which are connected to a carbonyl group and a sulfonyl group, respectively. Moreover, the carbonyl and sulfonyl groups are connected by a methylene group. Thus, the structure of compound **3a** can also be aptly confirmed through the single crystal structure.

On the other hand, the molecular weights of these radicals in control experiments were obtained by high-resolution mass spectrometry (HR-MS), and the error between the tested value and the calculated value was within a reasonable range.

### 2.4. Mechanism Investigation

**Scheme 3:**
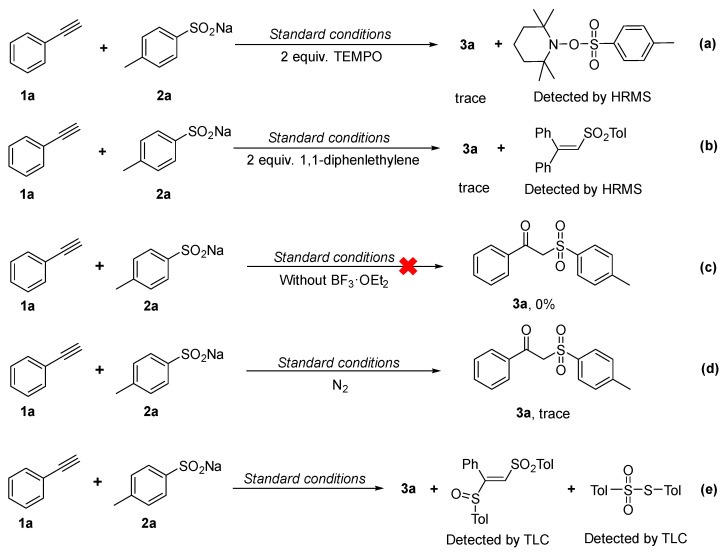
Control experiments.

In order to understand the reaction mechanism, some control experiments were carried out. The results are shown in Scheme 3.

Initially, once 2,2,6,6-tetramethyl-1-piperidinyloxy (TEMPO) as a free radical scavenger was added under the standard reaction conditions, the formation of **3a** was obviously suppressed (Scheme 3a). Importantly, the capture of the sulfonyl radical by TEMPO could be detected by HR-MS (Figure S2), indicating that a free radical pathway may be involved in this process.

**Figure 3:**
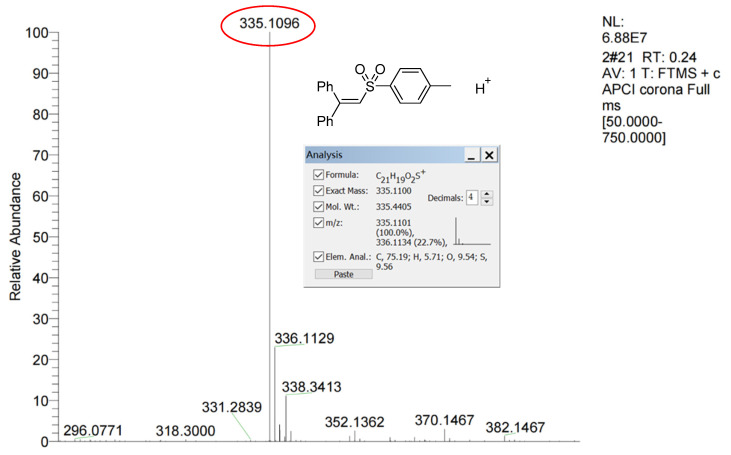
The HR-MS of the capture of sulfonyl radical by scavenger 1,1-diphenylethylene in the control experiment.

Similarly, when using 1,1-diphenylethylene as a free radical scavenger, there was no normal reaction for the formation of **3a** (Scheme 3b), and the capture of the sulfonyl radical by 1,1-diphenylethylene was also confirmed by HR-MS. In Figure 3, it can be seen that the theoretical calculated value of [M+H]^+^ of intermediates is 335.1100, and the actual test value is 335.1096 with an error value of 0.0004, which is indeed within the reasonable range. This also indicates that there is a free radical pathway.

What is more, the control experiment also implies that Lewis acid BF_3_·OEt_2_ could be essential for this reaction (Scheme 3c).

At the same time, in order to determine the source of the carboxyl oxygen atom in compound **3**, an experiment in an oxygen-free atmosphere was investigated. When the reaction was carried out in a N_2_ atmosphere, the target product could not be obtained (Scheme 3d). This result shows that O_2_ in the air may be the source of the carboxyl oxygen atom in the product [28].

Furthermore, when the reaction was carried out under standard conditions, β-sulfinyl alkenylsulfone and thiosulfonate were easily observed by TLC (Scheme 3e), which may be the byproducts of the reaction.

**Scheme 4:**
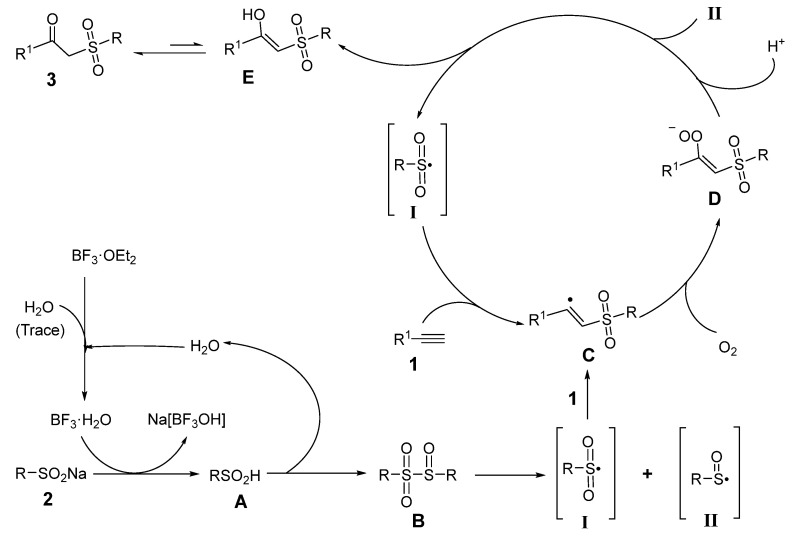
Proposed mechanism.

Thus, on the basis of the above control experiments and the previously reported studies [14,16,23,25,27,28,29,34,35,38,39], a possible reaction pathway is proposed as Scheme 4.

Firstly, BF_3_·H_2_O is produced in situ from BF_3_·OEt_2_ in the case of a trace amount of water [34,35]. Then, BF_3_·H_2_O reacts with sodium sulfinate **2**, giving sulfinic acid **A** and Na[BF_3_OH]. Subsequently, sulfinyl sulfone **B** is generated from sulfinic acid **A**, and it is easy to produce sulfonyl radical **I** and sulfinyl radical **II** from intermediate **B** under heating conditions [38,39].

Secondly, sulfonyl radical **I** is added to alkyne **1** to give intermediate **C** [14,23], which is then trapped by oxygen to generate intermediate **D** [25]. Finally, intermediate **D** forms intermediate **E** [16] via the hydrogen ion and radical **II**, while causing radical **II** to generate free radical **I**. Intermediate **E** is prone to tautomerism [27,28,29], giving product **3** (Scheme 4).

Considering the preliminary experimental results (Scheme 2a and 2b) [34,35], we speculate that the amount of BF_3_·OEt_2_ determines the amount of sodium sulfite involved in the reaction and less BF_3_·OEt_2_ is beneficial to the reaction of less sodium sulfite (Scheme 2c). At the same time, the reaction atmosphere [35] and the presence [34] or absence [35] of acetylene substrate determine which radicals are easier to generate and react. This is why different products can be produced in similar BF_3_·OEt_2_ catalytic systems (Scheme 2).

**Scheme 5:**
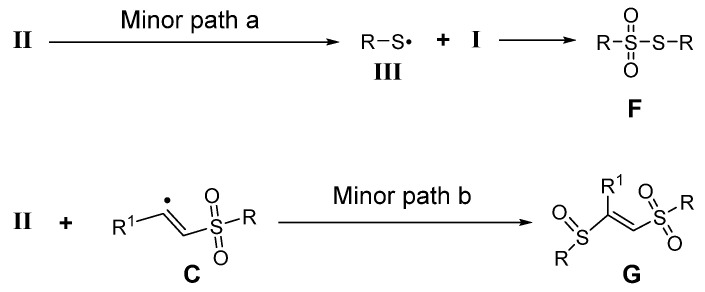
Proposed mechanism for the by-products.

In addition, as shown in Scheme 5, sulfinyl radicals **II** are extremely unstable in an air atmosphere and are prone to disproportionation to form sulfonyl radicals **I** and thiyl radicals **III** [40,41], which easily couple to form thiosulfonates **F** (**Minor path a** from intermediate **II**) [42,43]. Similarly, a small amount of β-sulfinyl alkenylsulfones **G** (**Minor path b** from intermediate **II**) [34] as a by-product can also be observed, which may be caused by the incomplete disproportionation of sulfinyl radicals [44].

### 2.5. Gram-scale Reaction

**Scheme 6:**
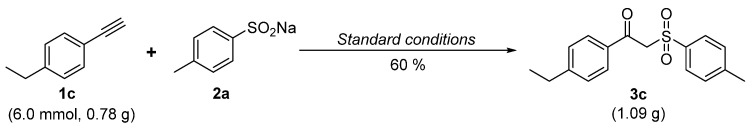
Gram-scale reaction of **3c**.

Considering that there is a wide application of β-keto sulfones in organic synthesis and biomedicine [45], in order to demonstrate the practicability of this reaction, further gram-scale research was carried out by selecting the synthesis of the target compound **3c** as an example (Scheme 6).

Obviously, the gram-scale synthesis of **3c** is satisfactory. With the increase in the amount of reactant **1c** from 0.3 to 6.0 mmol, the yield of **3c** is only slightly decreased (60% vs. 63%), and the target compound **3c** can be smoothly synthesized in gram-scale with a good yield.

## 3. Materials and Methods

### 3.1. General Information

^1^H and ^13^C NMR spectra were collected on an AVANCE NEO-600 in CDCl_3_ using tetramethylsilane (TMS) as an internal standard. Mass spectra were recorded on a Thermo Scientific ISQ gas chromatograph-mass spectrometer. High-resolution mass spectra (HR-MS) were obtained with a MAT 95XP mass spectrometer. The melting point (m.p.) was measured with a WRS-1B melting point instrument. Single-crystal X-ray analysis was performed using Agilent Gemini E. Reactions were monitored using thin-layer chromatography (TLC) and visualized with a UV light at 254 nm.

All reagents and solvents were purchased from commercial sources and used without further purification.

### 3.2. Experimental Procedure for Sodium Sulfinates **2**

**Scheme 7:**
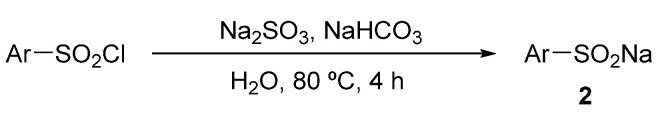
Synthesis route of different sodium sulfinates **2**.

Different sodium sulfinates **2** were synthesized as Scheme 7 according to the procedure in the literature [22,26,46].

According to the literature [22,26,46], the mixture of arylsulfonyl chloride (10 mmol), sodium sulfite **2** (20 mmol), and sodium bicarbonate (20 mmol) in H_2_O (15 mL) was stirred at 80 °C for 4 h. Water was removed by a rotary evaporator. Then, the remaining solid was extracted and recrystallized by ethanol to obtain the required compound **2**.

### 3.3. Experimental Procedure for Compounds **3a**-**3t**

**Scheme 8:**
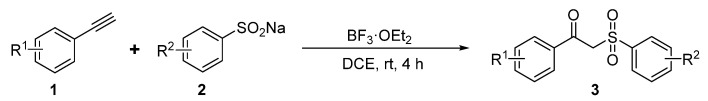
Synthesis route for Compounds **3a**-**3t**.

As shown in Scheme 8, the mixture of alkyne compound **1** (0.30 mmol, 1.0 equiv.), sodium sulfinate **2** (0.72 mmol, 2.4 equiv.), and BF_3_·OEt_2_ (0.15 mmol, 0.5 equiv.) in DCE (4 mL) under an air atmosphere was stirred at room temperature for 4 h. After the completion of the reaction, ethyl acetate (EA) (15 mL) was poured into the reaction mixture. The organic layers were extracted with a saturated sodium chloride solution (3 × 15 mL). Then, the organic layer was dried over anhydrous Na_2_SO_4_. Finally, after the filtration and evaporation of the solvents under reduced pressure, the crude product was purified by column chromatography on silica gel to afford the desired product **3**.

### 3.4. Characterization Data for All Products **3a-3t**

The structures of the serial compounds **3a-3t** were systematically characterized via NMR, m.p., etc., and the corresponding data are summarized as follows:

(1) 1-Phenyl-2-tosylethan-1-one (**3a**), white solid (43.0 mg, 56%); m.p.: 106–108 °C (103–105 °C [23]); ^1^H NMR (600 MHz, CDCl_3_), *δ*, ppm: 2.44 (*s*, 3H, CH_3_), 4.72 (*s*, 2H, CH_2_), 7.33 (*d*, *J* = 8.4 Hz, 2H, ArH), 7.46–7.49 (*m*, 2H, ArH), 7.61 (*t*, *J* = 7.2 Hz, 1H, ArH), 7.76 (*d*, *J* = 8.4 Hz, 2H, ArH), 7.94 (*d*, *J* = 7.2 Hz, 2H); ^13^C NMR (150 MHz, CDCl_3_), *δ*, ppm: 21.9, 63.8, 128.8, 129.0, 129.5, 130.0, 134.5, 135.8, 135.9, 145.5, 188.3.

(2) 1-(*p*-Tolyl)-2-tosylethan-1-one (**3b**), white solid (50.1 mg, 58%); m.p.: 109–111 °C (119–121 °C [47]); ^1^H NMR (600 MHz, CDCl_3_), *δ*, ppm: 2.42 (*s*, 3H, CH_3_), 2.44 (*s*, 3H, CH_3_), 4.69 (*s*, 2H, CH_2_), 7.27 (*d*, *J* = 8.4 Hz, 2H, ArH), 7.33 (*d*, *J* = 8.4 Hz, 2H, ArH), 7.75 (*d*, *J* = 8.4 Hz, 2H, ArH), 7.85 (*d*, *J* = 8.4 Hz, 2H, ArH); ^13^C NMR (150 MHz, CDCl_3_), *δ*, ppm: 21.9, 21.9, 63.7, 128.7, 129.6, 129.7, 129.9, 133.5, 135.9, 145.4, 145.7, 187.8.

(3) 1-(4-Ethylphenyl)-2-tosylethan-1-one (**3c**), white solid (57.1 mg, 63%); m.p.: 95–97 °C (84% [48]); ^1^H NMR (600 MHz, CDCl_3_), *δ*, ppm: 1.26 (*t*, *J* = 7.2 Hz, 3H, CH_3_), 2.44 (*s*, 3H, CH_3_), 2.72 (*q*, *J* = 7.2 Hz, 2H, CH_2_), 4.69 (*s*, 2H, CH_2_), 7.30 (*d*, *J* = 8.4 Hz, 2H, ArH), 7.33 (*d*, *J* = 8.4 Hz, 2H, ArH), 7.76 (*d*, *J* = 8.4 Hz, 2H, ArH), 7.87 (*d*, *J* = 8.4 Hz, 2H, ArH); ^13^C NMR (150 MHz, CDCl_3_), *δ*, ppm: 15.2, 21.8, 29.2, 63.7, 128.5, 129.7, 129.7, 129.9, 133.7, 135.9, 145.4, 151.8, 187.8.

(4) 1-(4-(*tert*-Butyl)phenyl)-2-tosylethan-1-one (**3d**), white solid (49.5 mg, 50%); m.p.: 93–94 °C (95–97 °C [49]); ^1^H NMR (600 MHz, CDCl_3_), *δ*, ppm: 1.34 (*s*, 9H, CH_3_), 2.43 (*s*, 3H CH_3_), 4.69 (*s*, 2H, CH_2_), 7.32 (*d*, *J* = 8.4 Hz, 2H, ArH), 7.48 (*d*, *J* = 8.4 Hz, 2H, ArH), 7.76 (*d*, *J* = 8.4 Hz, 2H, ArH), 7.88 (*d*, *J* = 8.4 Hz, 2H, ArH); ^13^C NMR (150 MHz, CDCl_3_), *δ*, ppm: 21.8, 31.1, 35.4, 63.6, 126.0, 128.7, 129.5, 129.9, 133.4, 135.9, 145.4, 158.5, 187.8.

(5) 1-(4-Fluorophenyl)-2-tosylethan-1-one (**3e**), white solid (33.3 mg, 38%); m.p.: 134–135 °C (125–127 °C [47]); ^1^H NMR (600 MHz, CDCl_3_), *δ*, ppm: 2.45 (*s*, 3H, CH_3_), 4.68 (*s*, 2H CH_2_), 7.14–7.17 (*m*, 2H, ArH), 7.34 (*d*, *J* = 8.4 Hz, 2H, ArH), 7.75 (*d*, *J* = 8.4 Hz, 2H, ArH), 7.99–8.01 (*m*, 2H, ArH); ^13^C NMR (150 MHz, CDCl_3_), *δ*, ppm: 21.9, 63.9, 116.2 (*d*, *J* = 22.5 Hz) 128.7, 130.0, 132.3, 132.4 (*d*, *J* = 10.5 Hz), 135.7, 145.7, 166.6 (*d*, *J* = 256.5 Hz), 186.7; ^19^F NMR (564 MHz, CDCl_3_), δ, ppm: -102.4.

(6) 1-(4-Chlorophenyl)-2-tosylethan-1-one (**3f**), white solid (44.4 mg, 48%); m.p.: 132–134 °C (138–139 °C [46]); ^1^H NMR (600 MHz, CDCl_3_), *δ*, ppm: 2.45 (*s*, 3H, CH_3_), 4.68 (*s*, 2H, CH_2_), 7.34 (*d*, *J* = 7.8 Hz, 2H, ArH), 7.46 (*d*, *J* = 8.4 Hz, 2H, ArH), 7.74 (*d*, *J* = 7.8 Hz, 2H, ArH), 7.90 (*d*, *J* = 8.4 Hz, 2H, ArH); ^13^C NMR (150 MHz, CDCl_3_), *δ*, ppm: 21.9, 63.9, 128.7, 129.3, 130.0, 130.9, 134.2, 135.7, 141.2, 145.7, 187.2.

(7) 1-(4-Bromophenyl)-2-tosylethan-1-one (**3g**), white solid (53.9 mg, 51%); m.p.: 140–141 °C (143–145 °C [23]); ^1^H NMR (600 MHz, CDCl_3_), *δ*, ppm: 2.45 (*s*, 3H, CH_3_), 4.67 (*s*, 2H, CH_2_), 7.34 (*d*, *J* = 8.4 Hz, 2H, ArH), 7.63 (*d*, *J* = 9.0 Hz, 2H, ArH), 7.74 (*d*, *J* = 8.4 Hz, 2H, ArH), 7.82 (*d*, *J* = 9.0 Hz, 2H, ArH); ^13^C NMR (150 MHz, CDCl_3_), *δ*, ppm: 21.9, 63.9, 128.7, 130.0, 130.1, 131.0, 132.4, 134.6, 135.7, 145.7, 187.4.

(8) 1-(4-Nitrophenyl)-2-tosylethan-1-one (**3h**), white solid (30.6 mg, 32%); m.p.: 140–142 °C (145–147 °C [19]); ^1^H NMR (600 MHz, CDCl_3_), *δ*, ppm: 2.47 (*s*, 3H, CH_3_), 4.75 (*s*, 2H, CH_2_), 7.37 (*d*, *J* = 8.4 Hz, 2H, ArH), 7.74 (*d*, *J* = 8.4 Hz, 2H, ArH), 8.15 (*d*, *J* = 9.0 Hz, 2H, ArH), 8.33 (*d*, *J* = 9.0 Hz, 2H, ArH); ^13^C NMR (150 MHz, CDCl_3_), *δ*, ppm: 21.9, 64.3, 124.1, 128.7, 130.2, 130.7, 135.5, 140.1, 146.1, 151.0, 187.1.

(9) 2-Tosyl-1-(4-trifluoromethylphenyl)ethan-1-one (**3i**), white solid (35.9 mg, 35%); m.p.: 134–136 °C (129–131 °C [23]); ^1^H NMR (600 MHz, CDCl_3_), *δ*, ppm: 2.45 (*s*, 3H, CH_3_), 4.73 (*s*, 2H, CH_2_), 7.34 (*d*, *J* = 8.4 Hz, 2H, ArH), 7.73–7.76 (*m*, 4H, ArH), 8.07 (*d*, *J* = 8.4 Hz, 2H, ArH); ^13^C NMR (150 MHz, CDCl_3_), *δ*, ppm: 21.9, 64.0, 123.5 (*q*, *J* = 271.5 Hz), 126.0 (*q*, *J* = 4.5 Hz128.7, 129.9, 130.1, 135.5 (*q*, *J* = 33.0 Hz), 135.6, 138.4, 145.9, 187.6; ^19^F NMR (564 MHz, CDCl_3_), δ, ppm: -63.3.

(10) 1-(*m*-Tolyl)-2-tosylethan-1-one (**3j**), white solid (43.2 mg, 50%); m.p.: 97–98 °C (97–99 °C [47]); ^1^H NMR (600 MHz, CDCl_3_), *δ*, ppm: 2.40 (*s*, 3H, CH_3_), 2.44 (*s*, 3H, CH_3_), 4.70 (*s*, 2H, CH_2_), 7.33 (*d*, *J* = 8.4 Hz, 2H, ArH), 7.35–7.38 (*m*, 1H, ArH), 7.42 (*d*, *J* = 7.8 Hz, 1H, ArH), 7.71 (*s*, 1H, ArH), 7.74 (*d*, *J* = 7.8 Hz, 1H, ArH), 7.76 (*d*, *J* = 8.4 Hz, 2H, ArH); ^13^C NMR (150 MHz, CDCl_3_), *δ*, ppm: 21.4, 21.9, 63.7, 126.8, 128.8, 128.9, 129.8, 129.9, 135.3, 136.0, 138.9, 145.5, 188.4.

(11) 1-(3-Methoxyphenyl)-2-tosylethan-1-one (**3k**), colorless oil (50.2 mg, 55%) ( 54% [47]); ^1^H NMR (600 MHz, CDCl_3_), *δ*, ppm: 2.44 (*s*, 3H, CH_3_), 3.84 (*s*, 3H, OCH_3_), 4.70 (*s*, 2H, CH_2_), 7.16 (*d*, *J* = 7.8 Hz, 1H, ArH), 7.34 (*d*, *J* = 8.4 Hz, 2H, ArH), 7.37–7.40 (*m*, 1H, ArH), 7.43 (*s*, 1H, ArH), 7.52 (*d*, *J* = 7.8 Hz, 1H, ArH), 7.77 (*d*, *J* = 8.4 Hz, 2H, ArH); ^13^C NMR (150 MHz, CDCl_3_), *δ*, ppm: 21.9, 55.6, 63.8, 113.1, 121.3, 122.3, 128.8, 130.0, 135.9, 137.2, 145.5, 160.1, 188.4.

(12) 1-(3-Chlorophenyl)-2-tosylethan-1-one (**3l**), white solid (38.8 mg, 42%); m.p.: 71–73 °C (71–73 °C [47]); ^1^H NMR (600 MHz, CDCl_3_), *δ*, ppm: 2.45 (*s*, 3H, CH_3_), 4.68 (*s*, 2H, CH_2_), 7.34 (*d*, *J* = 7.8 Hz, 2H, ArH), 7.42–7.46 (*m*, 1H, ArH), 7.58 (*d*, *J* = 7.2 Hz, 1H, ArH), 7.75 (*d*, *J* = 7.8 Hz, 2H, ArH), 7.84–7.86 (*m*, 2H, ArH); ^13^C NMR (150 MHz, CDCl_3_), *δ*, ppm: 21.9, 63.9, 127.7, 128.7, 129.3, 130.1, 130.3, 134.4, 135.4, 135.7, 137.4, 145.8, 187.2.

(13) 1-(3-Bromophenyl)-2-tosylethan-1-one (**3m**), white solid (49.6 mg, 47%); m.p.: 140–142 °C (140–141 °C [50]); ^1^H NMR (600 MHz, CDCl_3_), *δ*, ppm: 2.45 (*s*, 3H, CH_3_), 4.68 (*s*, 2H, CH_2_), 7.34 (*d*, *J* = 8.4 Hz, 2H, ArH), 7.36–7.39 (*m*, 1H, ArH), 7.72–7.75 (*m*, 3H, ArH), 7.89 (*d*, *J* = 7.8 Hz, 1H, ArH), 8.00 (*s*, 1H, ArH); ^13^C NMR (150 MHz, CDCl_3_), *δ*, ppm: 21.9, 63.8, 123.3, 128.2, 128.7, 130.1, 130.5, 132.2, 135.6, 137.3, 137.5, 145.8, 187.1.

(14) 1-(*o*-Tolyl)-2-tosylethan-1-one (**3n**), white solid (34.6 mg, 40%); m.p.: 99–101 °C (95–97 °C [51]); ^1^H NMR (600 MHz, CDCl_3_), *δ*, ppm: 2.43 (*s*, 3H, CH_3_), 2.44 (*s*, 3H, CH_3_), 4.69 (*s*, 2H, CH_2_), 7.24–7.26 (*m*, 1H, ArH), 7.28 (*d*, *J* = 7.8 Hz, 1H, ArH), 7.33 (*d*, *J* = 8.4 Hz, 2H, ArH), 7.41–7.43 (*m*, 1H, ArH), 7.73 (*d, J* = 7.8 Hz, 1H, ArH), 7.75 (*d*, *J* = 8.4 Hz, 2H, ArH); ^13^C NMR (150 MHz, CDCl_3_), *δ*, ppm: 21.7, 21.9, 65.7, 126.0, 128.6, 130.0, 130.6, 132.4, 132.9, 135.8, 136.1, 140.2, 145.4, 190.6.

(15) 1-(2-Methoxyphenyl)-2-tosylethan-1-one (**3o**), white solid (44.7 mg, 49%); m.p.: 124–126 °C (128–130 °C [51]); ^1^H NMR (600 MHz, CDCl_3_), *δ*, ppm: 2.42 (*s*, 3H, CH_3_), 3.88 (*s*, 3H, OCH_3_), 4.92 (*s*, 2H, CH_2_), 6.90 (*d*, *J* = 8.4 Hz, 1H, ArH), 6.97–7.00 (*m*, 1H, ArH), 7.29 (*d*, *J* = 8.4 Hz, 2H, ArH), 7.47–7.50 (*m*, 1H, ArH), 7.65 (*d*, 1H, *J* = 7.8 Hz, ArH), 7.74 (*d*, *J* = 8.4 Hz, 2H, ArH); ^13^C NMR (150 MHz, CDCl_3_), *δ*, ppm: 21.7, 55.7, 67.5, 111.7, 121.0, 126.3, 128.5, 129.5, 131.3, 135.2, 136.7, 144.8, 159.0, 189.2.

(16) 1-(Naphthalen-2-yl)-2-tosylethan-1-one (**3p**), white solid (44.7 mg, 46%); m.p.: 150–151 °C (150–152 °C [17]); ^1^H NMR (600 MHz, CDCl_3_), *δ*, ppm: 2.43 (*s*, 3H, CH_3_), 4.86 (*s*, 2H, CH_3_), 7.34 (*d*, *J* = 8.4 Hz, 2H, ArH), 7.59–7.62 (*m*, 1H, ArH), 7.65–7.68 (*m*, 1H, ArH), 7.80 (*d*, *J* = 8.4 Hz, 2H, ArH), 7.90–7.93 (*m*, 2H, ArH), 7.98–8.00 (*m*, 2H, ArH), 8.48 (*s*, 1H, ArH); ^13^C NMR (150 MHz, CDCl_3_), *δ*, ppm: 21.8, 64.0, 124.1, 127.3, 128.0, 128.8, 129.0, 129.5, 130.0, 130.1, 132.3, 132.4, 133.3, 135.8, 135.2, 145.6, 188.4

(17) 1-Phenyl-2-(phenylsulfonyl)ethan-1-one (**3q**), white solid (39.8 mg, 51%); m.p.: 86–88 °C (85–87 °C [23]); ^1^H NMR (600 MHz, CDCl_3_), *δ*, ppm: 4.74 (*s*, 2H, CH_2_), 6.46–7.49 (*m*, 2H, ArH), 7.53–7.56 (*m*, 2H, ArH-11,13), 7.62 (*t*, *J* = 7.8 Hz, 1H, ArH), 7.66 (*t*, *J* = 7.8 Hz, 1H, ArH), 7.90 (*d*, *J* = 7.2 Hz, 2H, ArH), 7.93 (*d*, *J* = 7.2 Hz, 2H, ArH); ^13^C NMR (150 MHz, CDCl_3_), *δ*, ppm: 63.6, 128.7, 129.0, 129.3, 129.4, 134.4, 134.5, 135.8, 138.8, 188.1.

(18) 2-(4-Methoxypheny)sulfonyl)-1-phenylethan-1-one (**3r**), white solid (50.6 mg, 53%); m.p.: 100–102 °C (100–102 °C [25]); ^1^H NMR (600 MHz, CDCl_3_), *δ*, ppm: 3.86 (*s*, 3H, OCH_3_), 4.71 (*s*, 2H, CH_2_), 6.97 (*d*, *J* = 9.0 Hz, 2H, ArH), 7.46–7.48 (*m*, 2H, ArH), 7.61 (*t, J* = 7.8 Hz, 1H, ArH), 7.79 (*d*, *J* = 9.0 Hz, 2H, ArH), 7.93 (*d*, *J* = 7.2 Hz, 2H, ArH); ^13^C NMR (150 MHz, CDCl_3_), *δ*, ppm: 55.8, 63.8, 114.5, 128.9, 129.4, 130.3, 131.0, 134.4, 135.9, 164.2, 188.4.

(19) 2-(4-Chlorophenylsulfonyl)-1-phenylethan-1-one (**3s**), white solid (44.1 mg, 50%); m.p.: 131–132 °C (132–134 °C [17]); ^1^H NMR (600 MHz, CDCl_3_), *δ*, ppm: 4.74 (*s,* 2H, CH_2_), 7.48–7.51 (*m*, 2H, ArH), 7.52 (*d*, *J* = 8.4 Hz, 2H, ArH), 7.64 (*t*, *J* = 7.8 Hz, 1H, ArH), 7.83 (*d*, *J* = 8.4 Hz, 2H, ArH), 7.94 (*d*, *J* = 7.8 Hz, 2H, ArH); ^13^C NMR (150 MHz, CDCl_3_), *δ*, ppm: 63.5, 129.1, 129.4, 129.7, 130.3, 134.7, 135.7, 137.2, 141.3, 188.0.

(20) 1-(4-Ethylphenyl)-2-phenylsulfonylethan-1-one (**3t**), white solid (53.6 mg, 62%); m.p.: 128–129 °C (127–129 °C [25]); ^1^H NMR (600 MHz, CDCl_3_), *δ*, ppm: 1.25 (*t*, *J* = 7.8 Hz, 3H, CH_3_), 2.71 (*q*, *J* = 7.8 Hz, 2H, CH_2_), 4.72 (*s*, 2H, CH_2_), 7.29 (*d*, *J* = 8.4 Hz, ArH), 7.52–7.55 (*m*, 2H, ArH), 7.65 (*t*, *J* = 7.8 Hz, 1H, ArH), 7.86 (*d*, *J* = 8.4 Hz, 2H, ArH), 7.89 (*d*, *J* = 7.2 Hz, 2H, ArH); ^13^C NMR (150 MHz, CDCl_3_), *δ*, ppm: 15.1, 29.1, 63.5, 128.5, 128.7, 129.3, 129.7, 133.6, 134.3, 138.9, 151.8, 187.6.

The detailed ^1^H, ^13^C, and ^19^F NMR spectra for all compounds **3a**-**3t** are provided in the Supplementary Materials.

## 4. Conclusions

In summary, we successfully found a BF_3_·OEt_2_-mediated oxysulfonylation reaction of sodium sulfinates and alkynes. The reaction does not need any metal catalysts, and the simple and mild conditions make it a convenient procedure for the synthesis of β-keto sulfones. A possible radical mechanism was also proposed on the basis of control studies. Importantly, this reaction uses oxygen in the air as an oxidant and does not need to use chemical reagents with pungent odors such as pyridine and acetic acid. It meets the requirements of the development of green chemistry.

## Figures and Tables

**Table 1:** Optimization of reaction conditions ^[a]^.

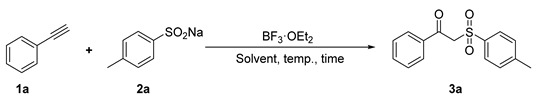						
**Entry**	**Acid (equiv.)**	**1a** **:2a**	**Temp. (** **°C)**	**Time (h)**	**Solvent**	**Yield (%) ^[b]^**
1	BF_3_·OEt_2_ (0.5)	1:2.4	rt	4	DCM	38
2	BF_3_·OEt_2_ (0.5)	1:2.4	rt	6	DCM	33
3	BF_3_·OEt_2_ (0.5)	1:2.4	rt	2	DCM	15
4	BF_3_·OEt_2_ (0.5)	1:2.4	40	4	DCM	32
5	BF_3_·OEt_2_ (0.5)	1:2.4	0	4	DCM	Trace
6	BF_3_·OEt_2_ (0.25)	1:2.4	rt	4	DCM	22
7	BF_3_·OEt_2_ (0.75)	1:2.4	rt	4	DCM	27
**8**	**BF_3_·OEt_2_ (0.5)**	**1:2.4**	**rt**	**4**	**DCE**	**56**
9	BF_3_·OEt_2_ (0.5)	1:2.4	rt	4	EA	24
10	BF_3_·OEt_2_ (0.5)	1:2.4	rt	4	DMSO	N.D. ^[c]^
11	BF_3_·OEt_2_ (0.5)	1:2.4	rt	4	CH_3_OH	N.D.
12	BF_3_·OEt_2_ (0.5)	1:2.4	rt	4	C_2_H_5_OH	N.D.
13	BF_3_·OEt_2_ (0.5)	1:2.4	rt	4	THF	N.D.
14	BF_3_·OEt_2_ (0.5)	1:2.4	rt	4	CH_3_CN	18
15	BF_3_·OEt_2_ (0.5)	1:2.4	rt	4	Toluene	N.D.
16	BF_3_·OEt_2_ (0.5)	1:2.4	rt	4	Acetone	N.D.
17	BF_3_·OEt_2_ (0.5)	1:2.4	rt	4	DMF	N.D.
18	BF_3_·OEt_2_ (0.5)	1:2.4	rt	4	AcOH	N.D.
19	BF_3_·OEt_2_ (0.5)	1:2.4	rt	4	1,4-Dioxane	N.D.
20	BF_3_·OEt_2_ (0.5)	1:2.4	rt	4	CHCl_3_	12
21	BF_3_·OEt_2_ (0.5)	1:2.4	rt	4	Ethyl ether	N.D.
22	BF_3_·OEt_2_ (0.5)	1:2.4	rt	4	Dimethoxyethane	N.D.
23	BF_3_·OEt_2_ (0.5)	1:2.0	rt	4	DCE	43
24	BF_3_·OEt_2_ (0.5)	1:2.8	rt	4	DCE	42

^[a]^ Reaction conditions: Usually, **1a** (0.3 mmol), **2a** (0.72 mmol), BF_3_∙OEt_2_ (0.15 mmol), and DCE (4 mL) at room temperature (rt) for 4 h under air atmosphere. ^[b]^ Isolated yield. ^[c]^ N.D. = Not Detected.

**Table 2:** Substrate scope of various alkyne compounds **1** sodium sulfinates **2** ^[a, b]^.

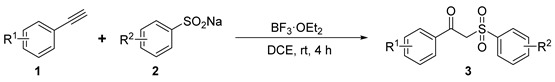
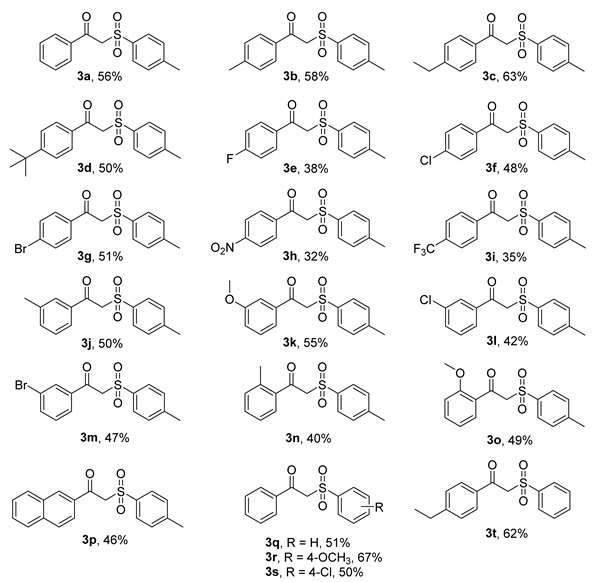

^[a]^ Reaction conditions: Usually, **1** (0.30 mmol), **2** (0.72 mmol), BF_3_·OEt_2_ (0.15 mmol), and DCE (4 mL) were added and stirred at room temperature (rt) for 4 h. ^[b]^ Isolated yield.

**Table 3:** X-ray crystal data and structure refinement for compound **3a**.

Compound	3a
Empirical formula	C_15_H_14_O_3_S
Formula weight	274.32
Temperature (K)	297
Wavelength (Å)	0.71073
Crystal system	monoclinic
Space group	P2_1_/n
Unit cell dimensions (Å, °)	*a* = 7.7540(5), *b* = 11.5019(8), *c* = 15.3137(10)
	*α* = 90, *β* = 98.746(7), *γ* = 90
Volume (Å^3^)	1349.88(16)
Z	4
Density (calculated) (g/cm^3^)	1.350
Absorption coefficient (mm^−1^)	0.240
F(000)	576.0
Theta range for data collection	2.691 to 29.072
Index ranges	−10 ≤ h ≤ 10, −15 ≤ k ≤ 13, −20 ≤ l ≤ 20
Reflections collected	6338
Independent reflections	3117 [R(int) = 0.0218, R(sigma) = 0.0379]
Completeness to theta = 29.072°	86.4 %
Absorption correction	Multi-Scan
Max. and min. transmission	1.000 and 0.885
Refinement method	Least Squares minimisation
Data / restraints / parameters	3117 / 0 / 173
Goodness-of-fit on F^2^	1.027
Final R indices [I>2sigma(I)]	R_1_ = 0.0489, wR_2_ = 0.1141
R indices (all data)	R_1_ = 0.0668, wR_2_ = 0.1229
Largest diff. peak and hole	0.26 and −0.26 e.Å-3

## Data Availability

All data supporting the findings of this study are available within the paper and within its Supplementary Materials published online.

## Supplementary Materials

The following supporting information can be downloaded at: https://www.mdpi.com/article/10.3390/molecules29153559/s1, and contains the ^1^H, ^13^C, and ^19^F NMR spectra for all compounds **3a**-**3t.**
