# Supplementary material for: Oxysulfonylation of Alkynes with Sodium Sulfinates to Access β-Keto Sulfones Catalyzed by BF3·OEt2
Source: Molecules. 2024 Jul 28;29(15):3559. doi: 10.3390/molecules29153559 (PMC11314596; doi:10.3390/molecules29153559)
Supplement: Supplementary file 1 [file molecules-29-03559-s001.zip › molecules-3126279-supplementary.pdf]

Supplementary Materials

Oxysulfonylation of alkynes with sodium sulfinates to access β-keto sulfones catalyzed by $BF_3·OEt_2$

Shi-Wei Yu[1], Zu-Jia Chen[1], Huan-Qing Li[1], Wen-Xi Li[1], Yun Li[1], Zong Li[1], Zhao-Yang Wang[1,*]

[1] School of Chemistry, South China Normal University, Guangzhou Key Laboratory of Analytical Chemistry for Biomedicine, GDMPA Key Laboratory for Process Control and Quality Evaluation of Chiral Pharmaceuticals, Key Laboratory of Theoretical Chemistry of Environment, Ministry of Education, Guangzhou 510006, P. R. China; 2021022671@m.scnu.edu.cn (S.-W.Y.); 2022022607@m.scnu.edu.cn (Z.-J.C.); 2022022611@m.scnu.edu.cn (H.-Q.L.); 2023022646@m.scnu.edu.cn (W.-X.L.); m15192479187@163.com (Y.L.); lzscnu@outlook.com (Z.L.); wangzy@scnu.edu.cn (Z.-Y.W.)

* Correspondence: wangzy@scnu.edu.cn (Z.-Y.W.).; Tel.: +86-020-3931-0258 (Z.-Y.W.); Fax: +86-020-3931-0187 (Z.-Y.W.)

Table of Contents

General Information....................[2]

Experimental Procedure for Sodium Sulfinates **2**....................[3]

Experimental Procedure for Compounds **3a-3t**....................[3]

Data of Single-crystal X-ray Analysis for **3a**....................[5]

Experimental Spectra Used in Discussions....................[6]

NMR Spectra for All Products **3a-3t**....................[7-27]

References....................[28]

General Information

^{1}H and ^{13}C NMR spectra were collected on an AVANCE NEO-600 in $CDCl_3$ using tetramethylsilane (TMS) as an internal standard. Mass spectra were recorded on a Thermo Scientific ISQ gas chromatograph-mass spectrometer. High-resolution mass spectra (HR-MS) were obtained with a MAT 95XP mass spectrometer. Melting point (m.p.) was measured with WRS-1B melting point instrument. Single-crystal X-ray analysis was obtained using Agilent Gemini E. Reactions were monitored using thin-layer chromatography (TLC) and visualized with UV light at 254 nm.

All reagents and solvents were purchased from commercial sources and used without further purification. Different sodium sulfinates **2** were synthesized according to the literature procedure [1].

Experimental Procedure for Sodium Sulfinates 2

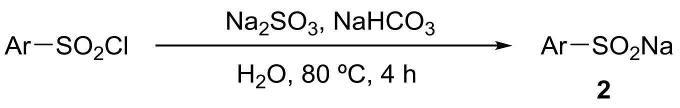

According to the literature [1], the mixture of arylsulfonyl chloride (10 mmol), sodium sulfite (20 mmol), sodium bicarbonate (20 mmol) in H_2O (15 mL) was stirred at 80 ° C for 4 h. Water was removed by rotary evaporator.

Then, the remaining solid was extracted and recrystallized by ethanol to get the required compound **2**.

Experimental Procedure for Compounds 3a-3t

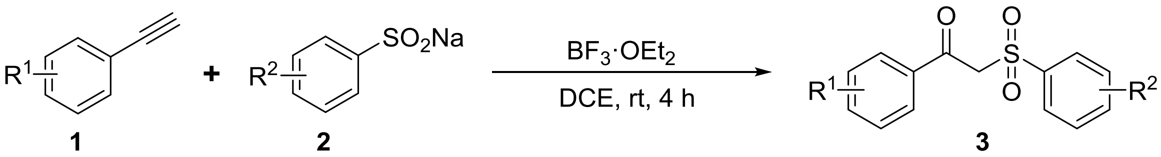

The mixture of alkyne compound **1** (0.30 mmol, 1.0 equiv.), sodium sulfinate **2** (0.72 mmol, 2.4 equiv.), and $BF_3{\cdot}OEt_2$ (0.15 mmol, 0.5 equiv.) in DCE (4 mL) under air atmosphere was stirred at room temperature for 4 h. After the completion of reaction, EtOAc (15 mL) was poured into the reaction mixture. The organic layers were extracted with the saturated sodium chloride solution (3 × 15 mL).

Then, the organic layer was dried over anhydrous Na_2SO_4. Finally, after the filtration and the evaporation of the solvents under reduced pressure, the crude product was purified by column chromatography on silica gel to afford the desired product **3**.

Data of Single-crystal X-ray Analysis

Table S1. Crystal data and structure refinement for **3a**.

Compound	3a
Empirical formula	$C_{15}H_{14}O_3S$
Formula weight	274.32
Temperature (K)	297
Wavelength (Å)	0.71073
Crystal system	monoclinic
Space group	$P2_1/n$
Unit cell dimensions (Å, °)	a = 7.7540(5), b = 11.5019(8), c = 15.3137(10)
	α = 90, β = 98.746(7), γ = 90
Volume (Å^3)	1349.88(16)
Z	4
Density (calculated) (g/cm^3)	1.350
Absorption coefficient (mm^{-1})	0.240
F(000)	576.0
Theta range for data collection	2.691 to 29.072
Index ranges	$-10 \le h \le 10$, $-15 \le k \le 13$, $-20 \le l \le 20$
Reflections collected	6338
Independent reflections	3117 [R(int) = 0.0218, R(sigma) = 0.0379]
Completeness to theta = 29.072°	86.4 %
Absorption correction	Multi-Scan
Max. and min. transmission	1.000 and 0.885
Refinement method	Least Squares minimisation
Data / restraints / parameters	3117 / 0 / 173
Goodness-of-fit on F^2	1.027
Final R indices [I>2sigma(I)]	R_1 = 0.0489, wR_2 = 0.1141
R indices (all data)	R_1 = 0.0668, wR_2 = 0.1229
Largest diff. peak and hole	0.26 and -0.26 e.Å^{-3}

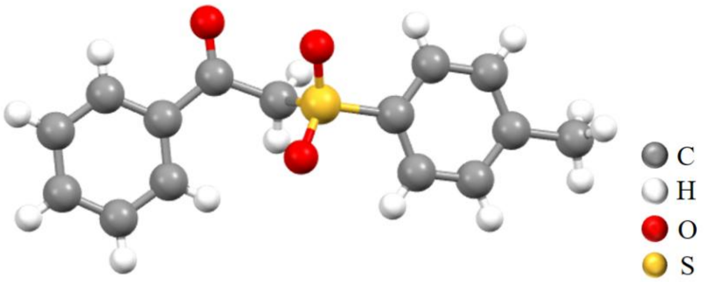

Figure S1. The molecular structure of **3a**.

Experimental Spectra Used in Discussions

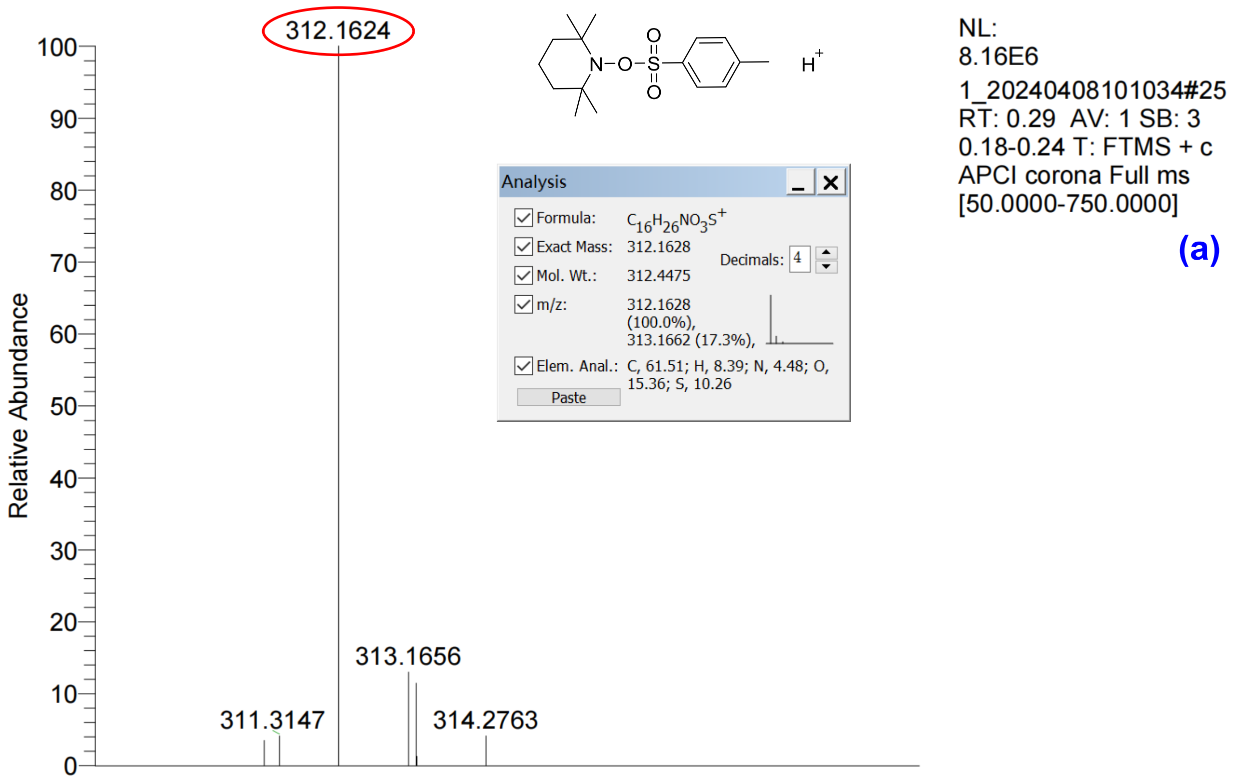

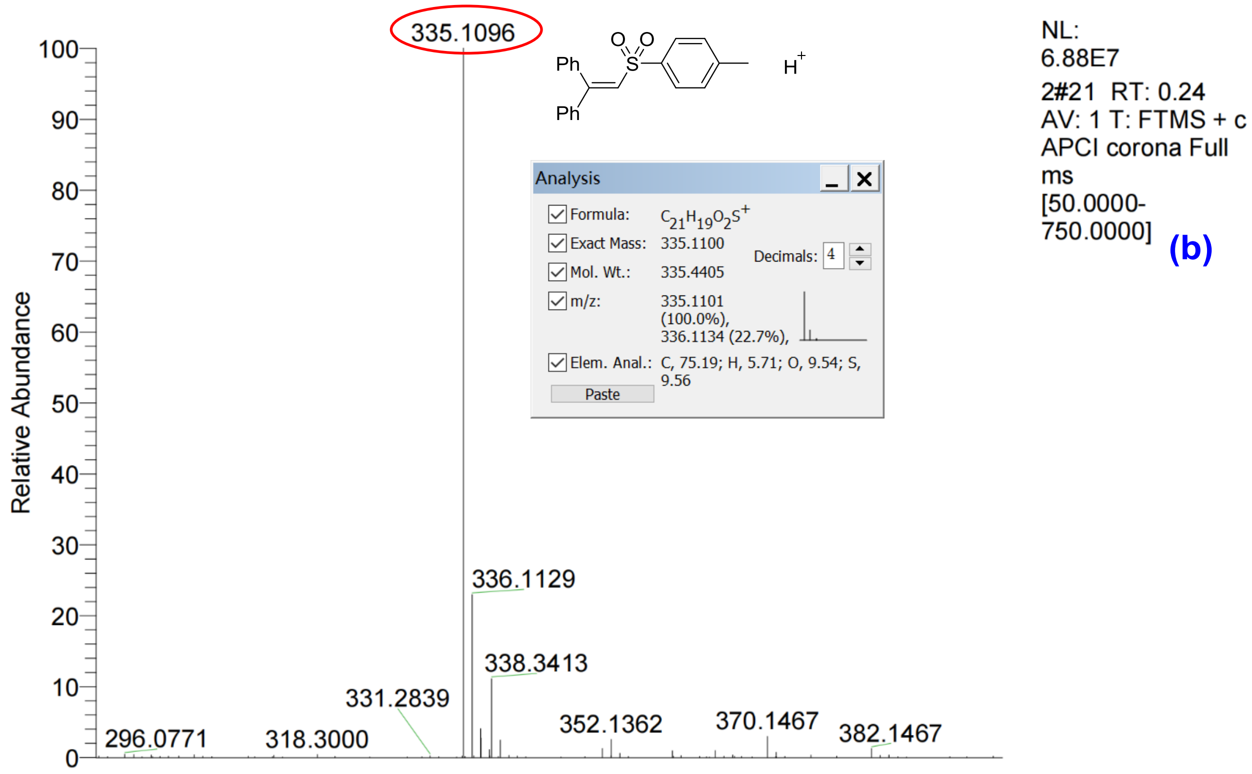

Figure S2. The HR-MS of the detected intermediates in the control experiments.

NMR Spectra for All Compounds 3a-3t

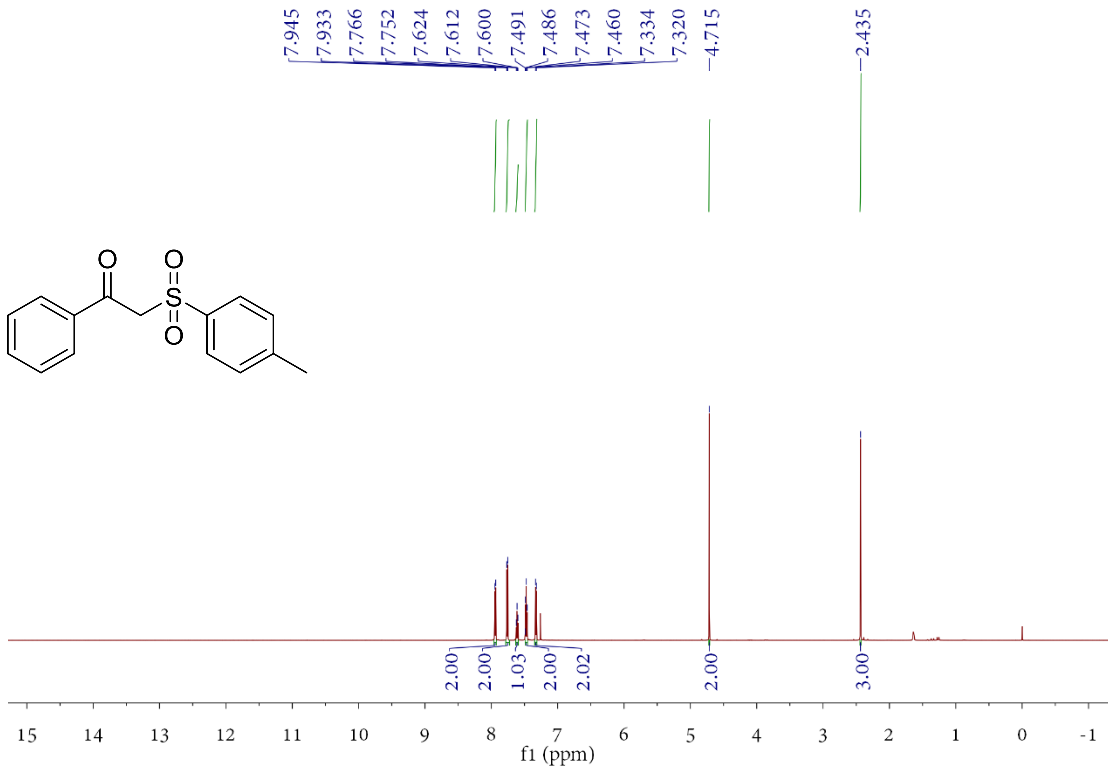

^{1}H NMR spectrum of compound **3a**

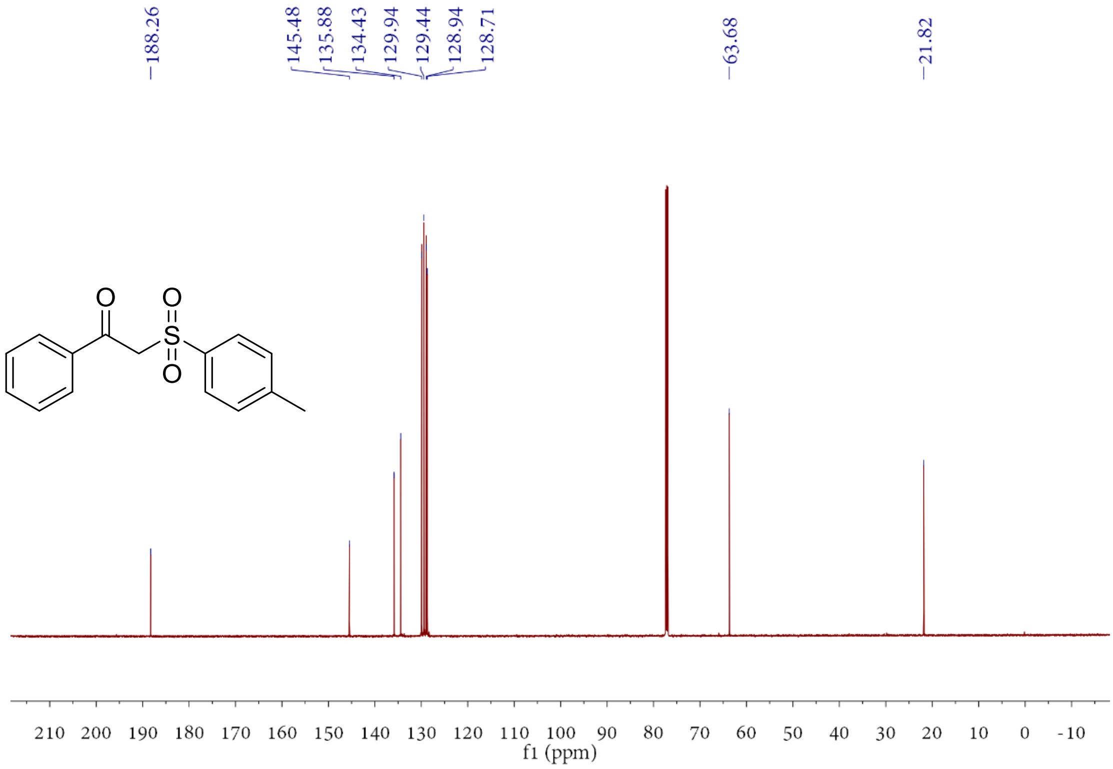

^{13}C NMR spectrum of compound **3a**

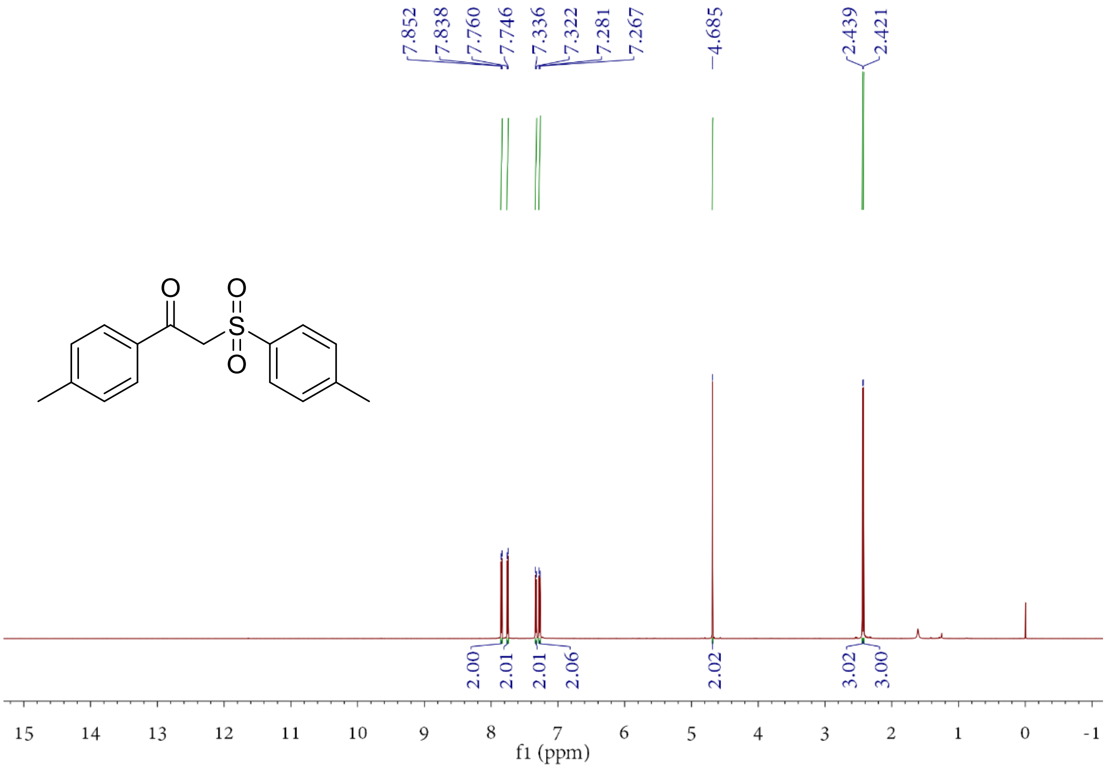

^{1}H NMR spectrum of compound **3b**

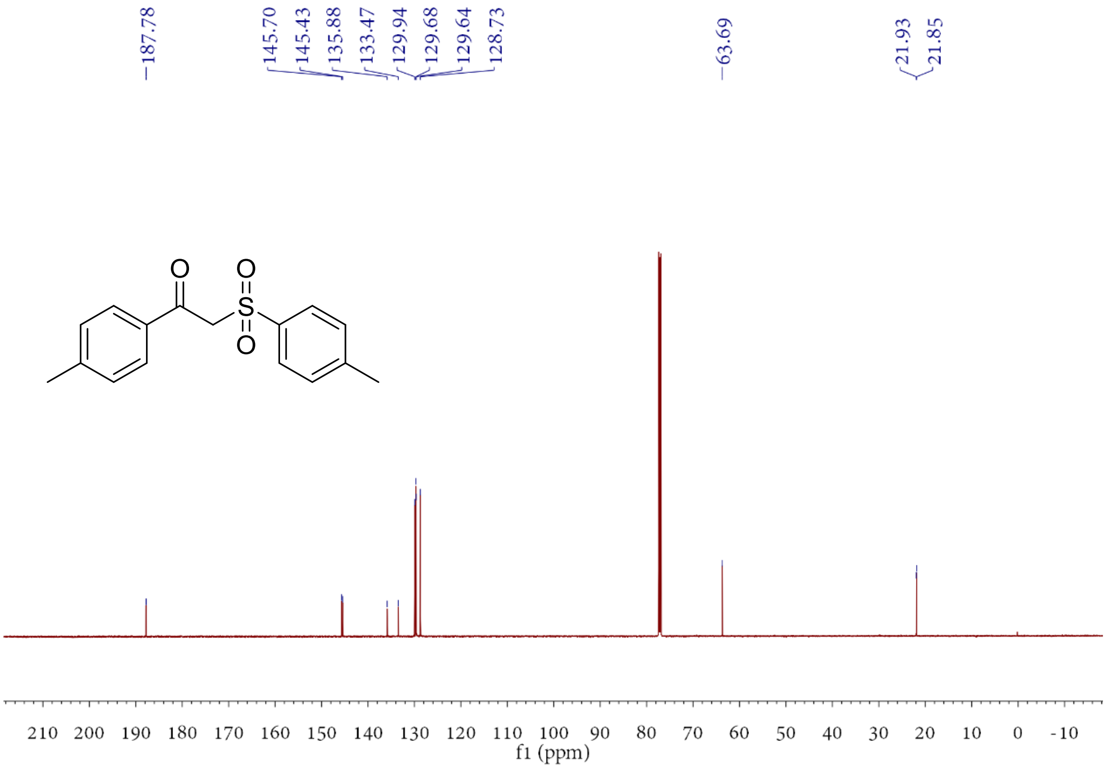

^{13}C NMR spectrum of compound **3b**

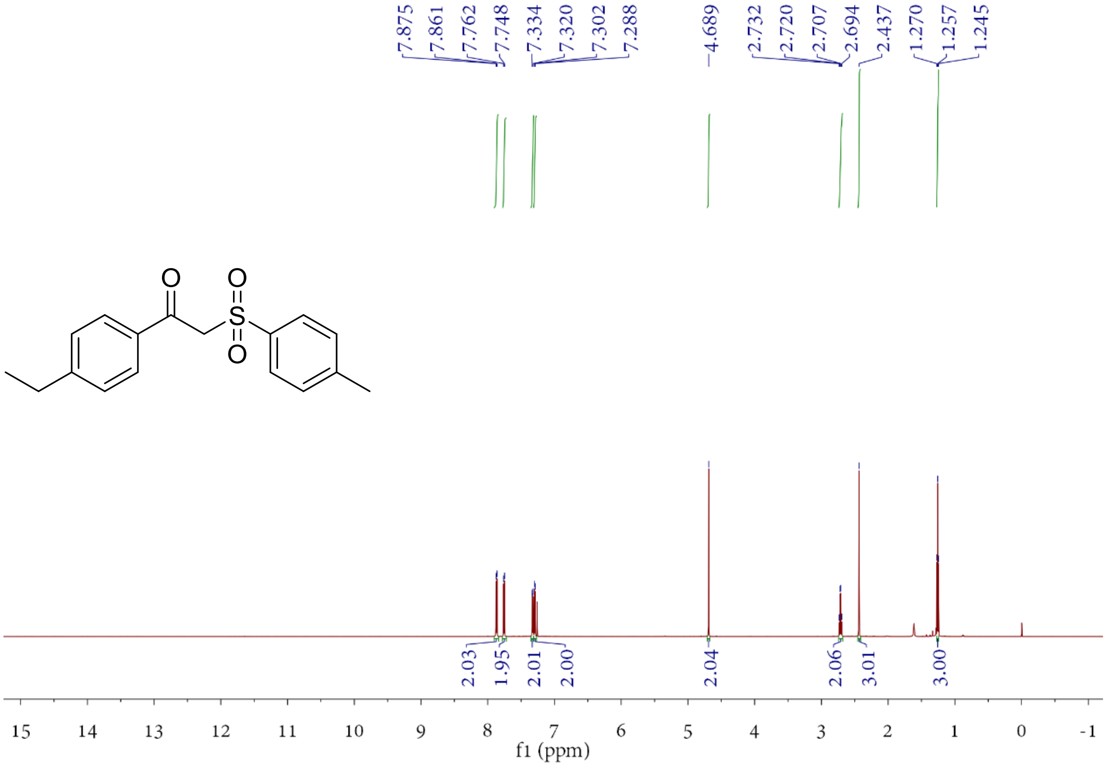

^{1}H NMR spectrum of compound **3c**

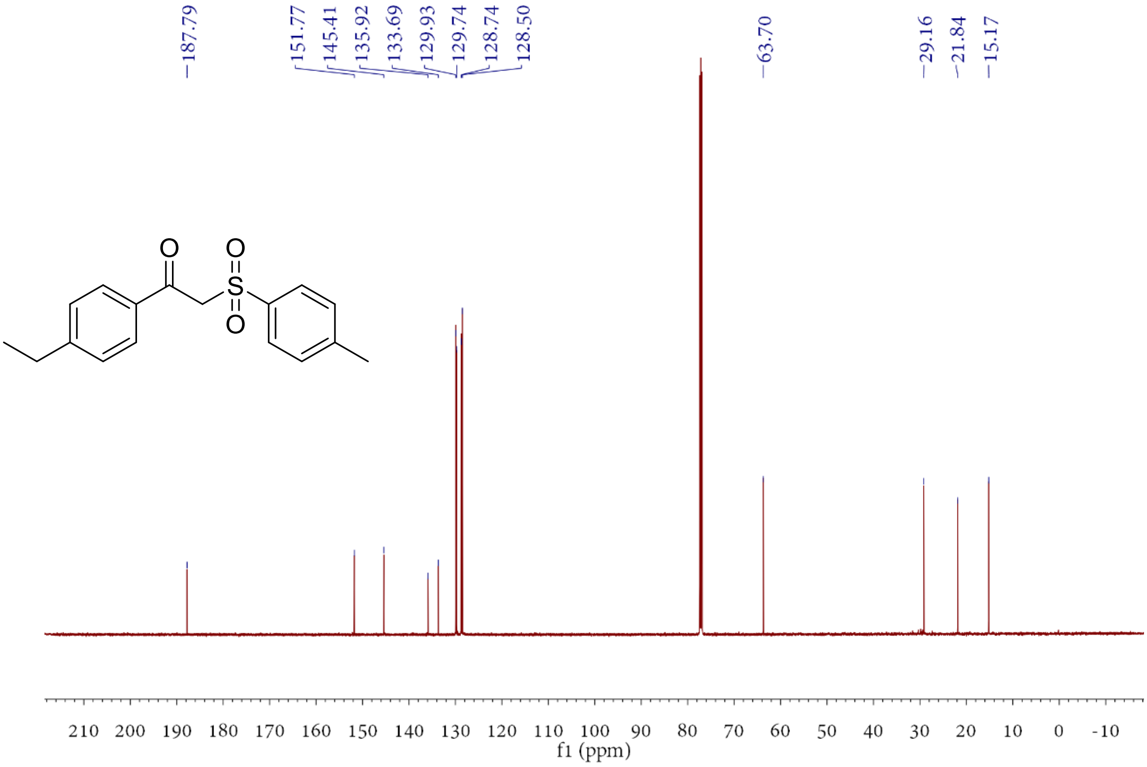

^{13}C NMR spectrum of compound **3c**

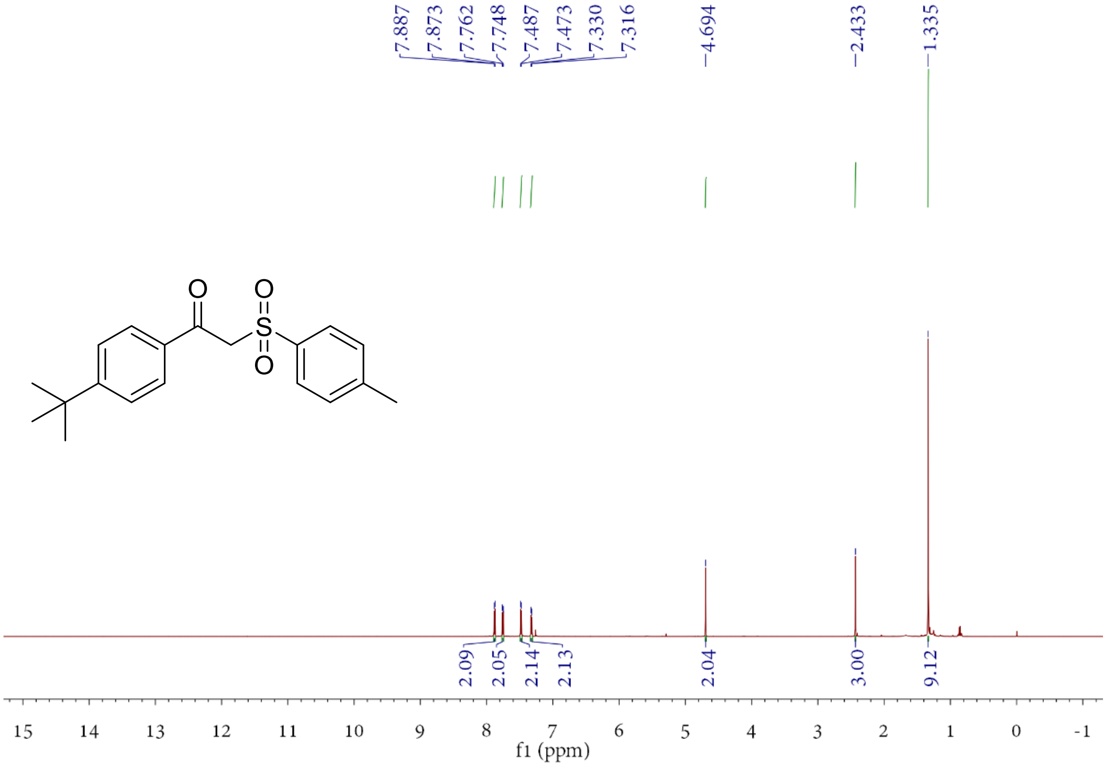

^{1}H NMR spectrum of compound **3d**

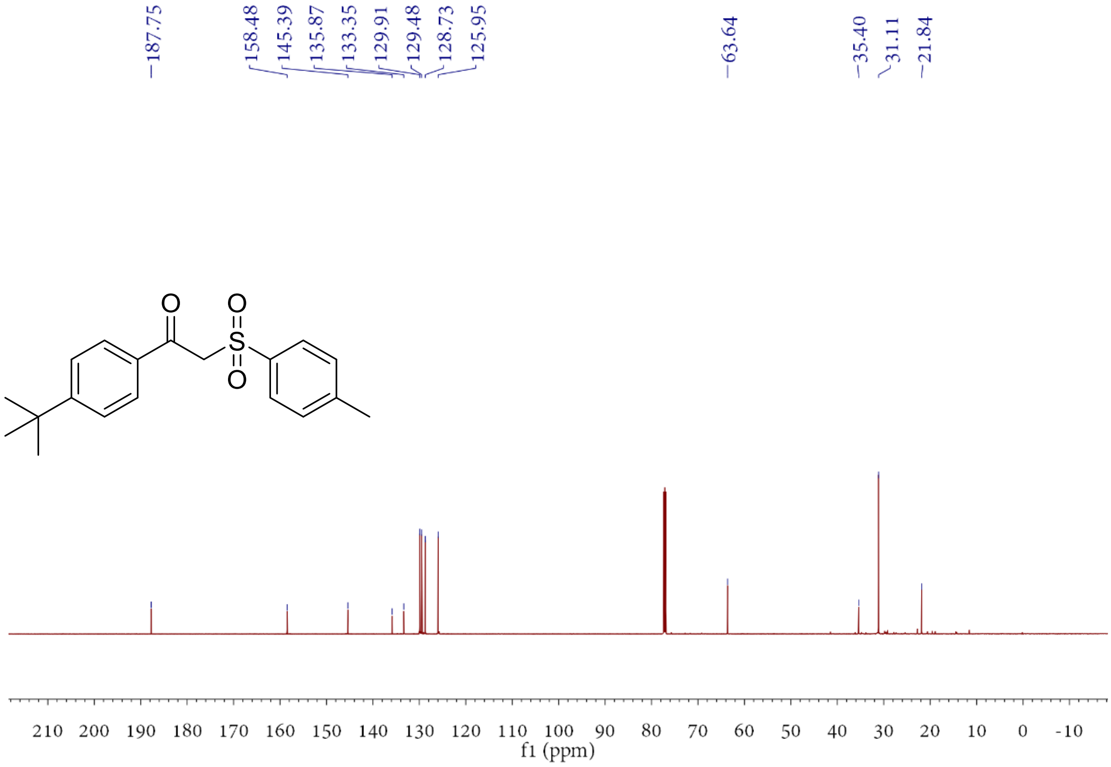

^{13}C NMR spectrum of compound **3d**

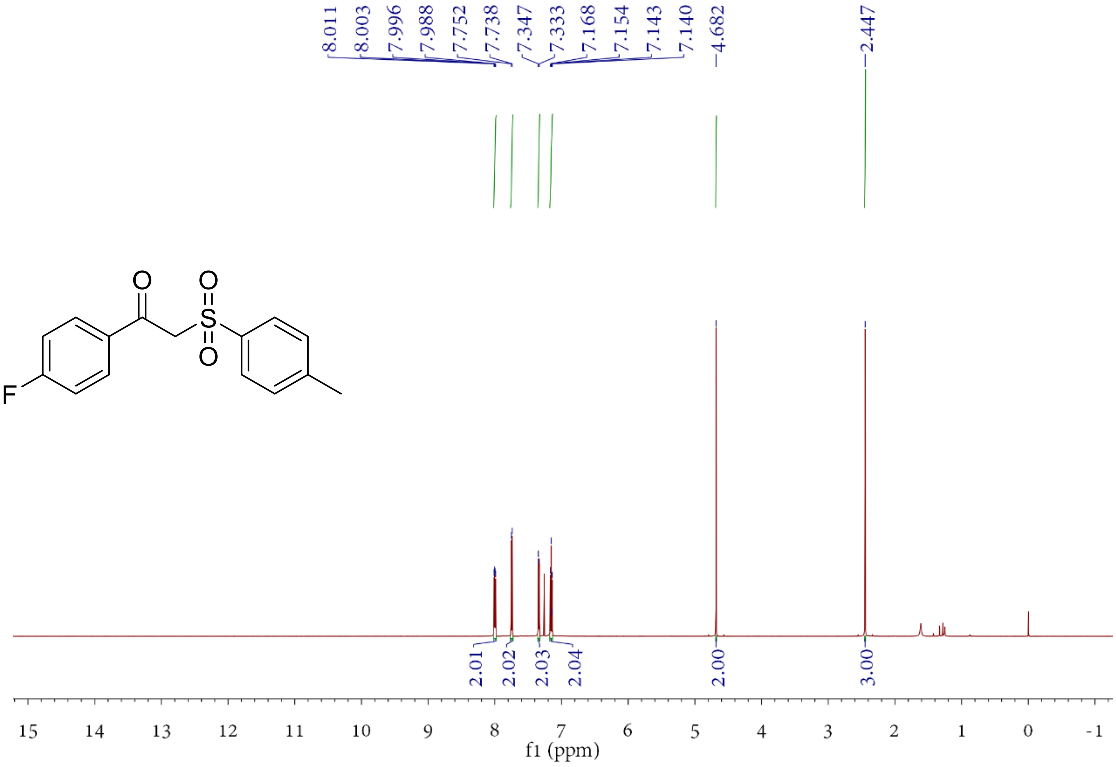

^{1}H NMR spectrum of compound **3e**

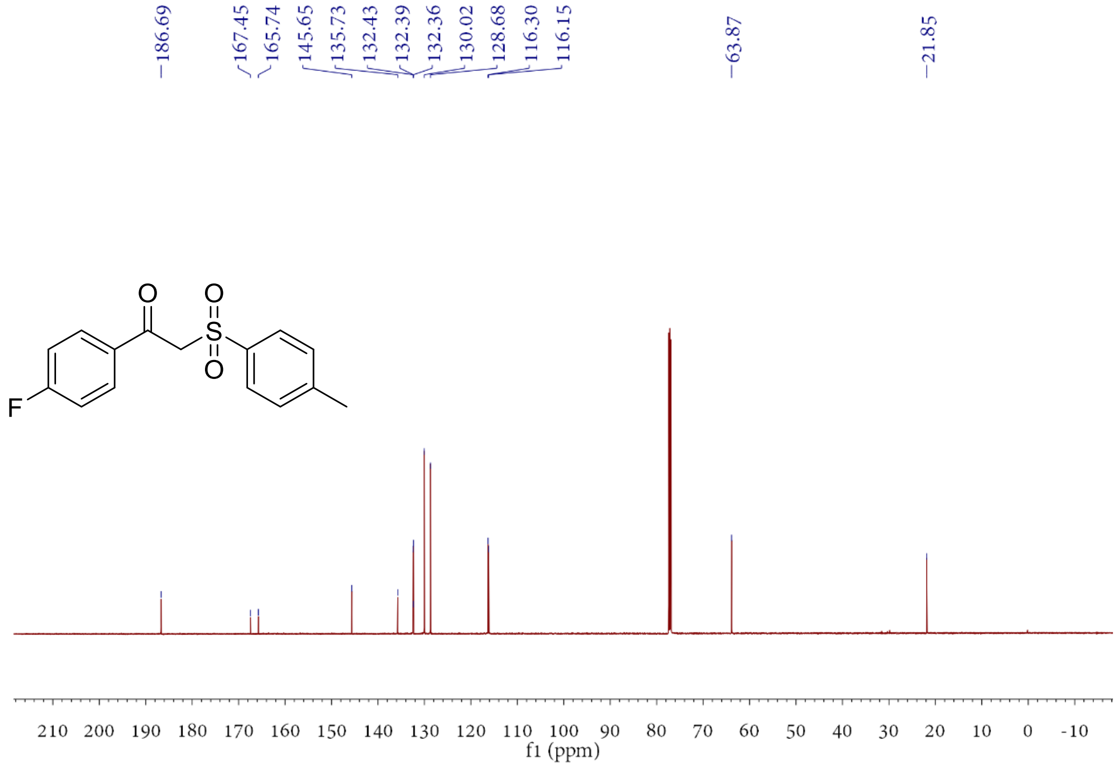

^{13}C NMR spectrum of compound **3e**

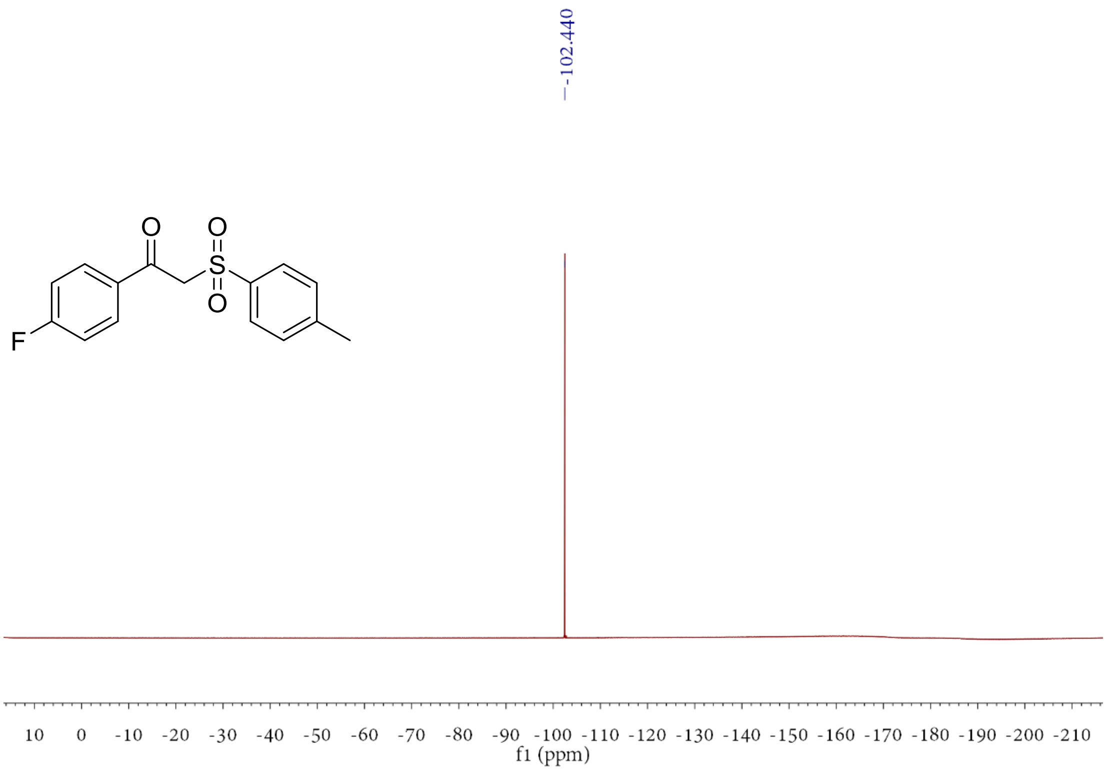

^{19}F NMR spectrum of compound **3e**

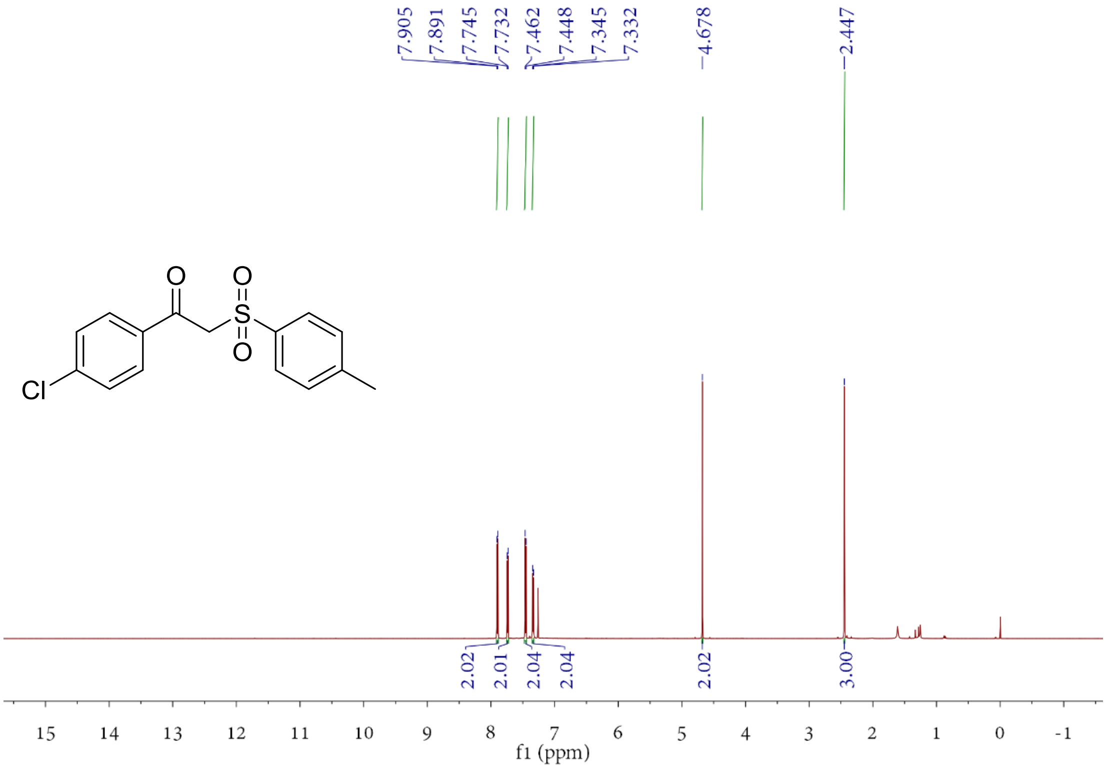

^{1}H NMR spectrum of compound **3f**

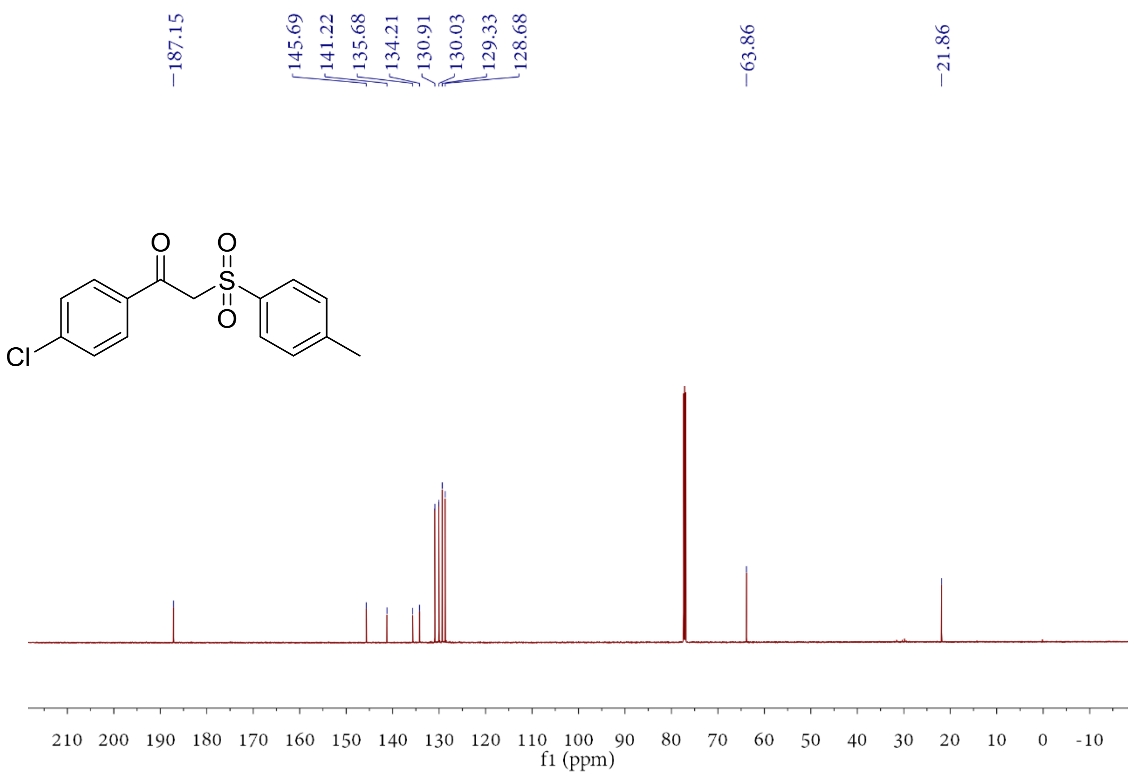

^{13}C NMR spectrum of compound **3f**

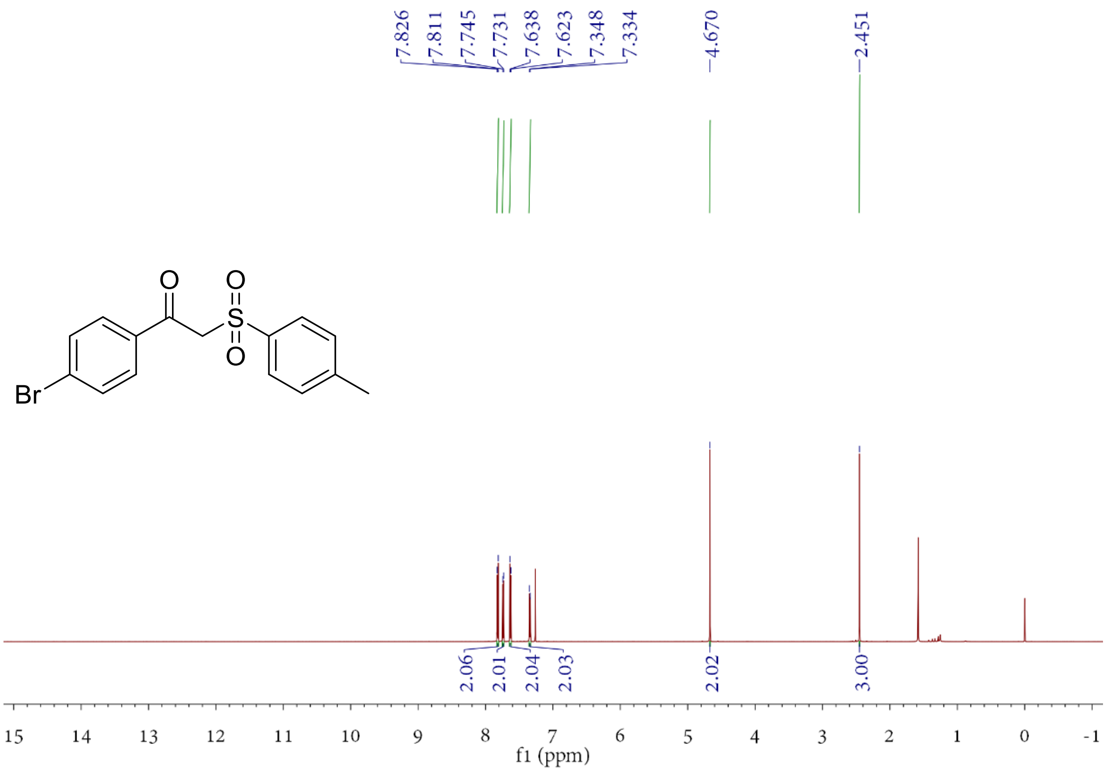

^{1}H NMR spectrum of compound **3g**

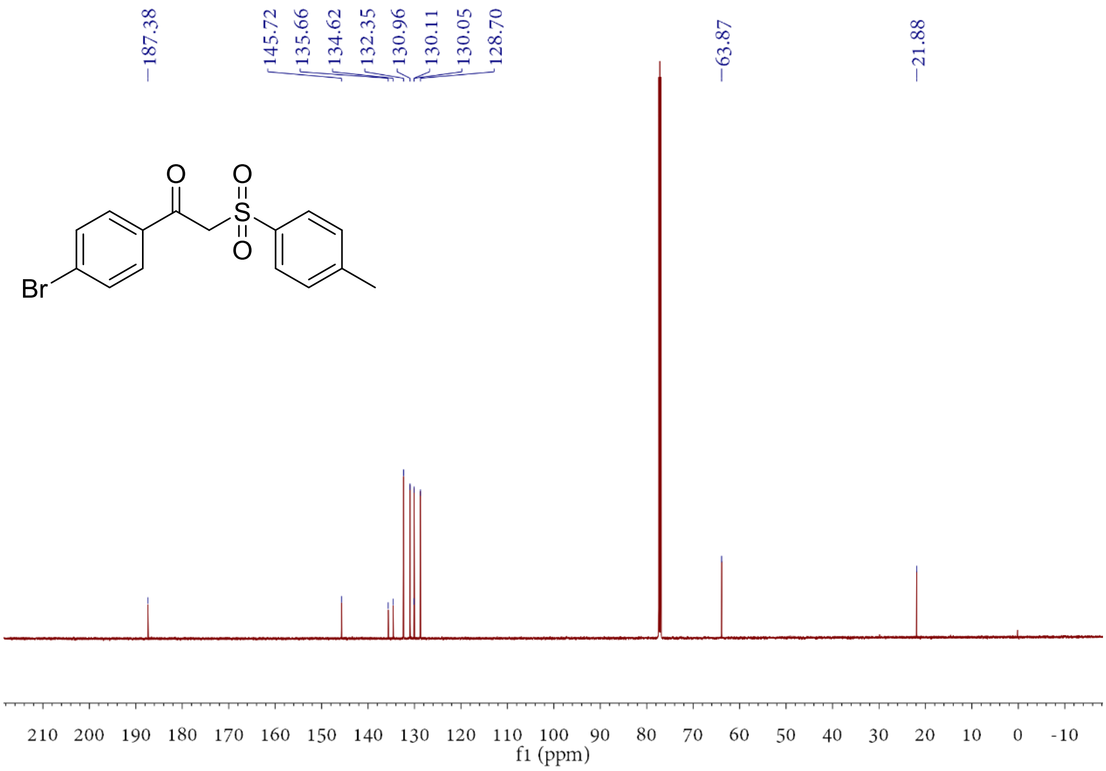

^{13}C NMR spectrum of compound **3g**

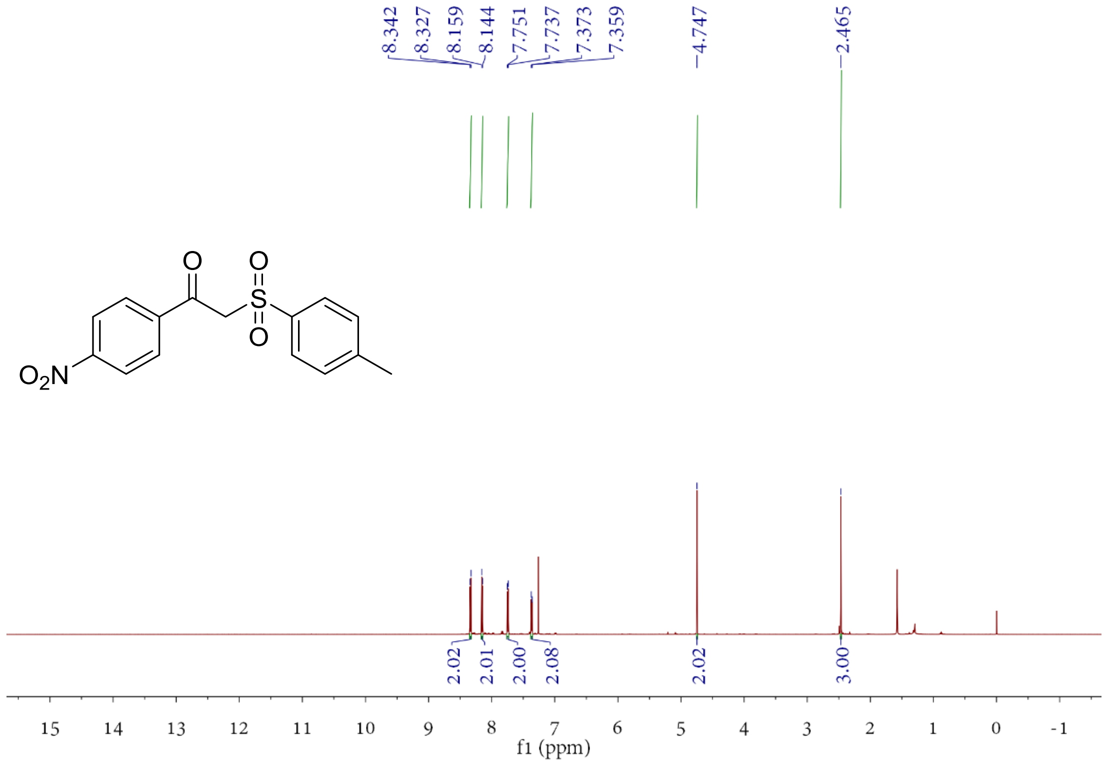

^{1}H NMR spectrum of compound **3h**

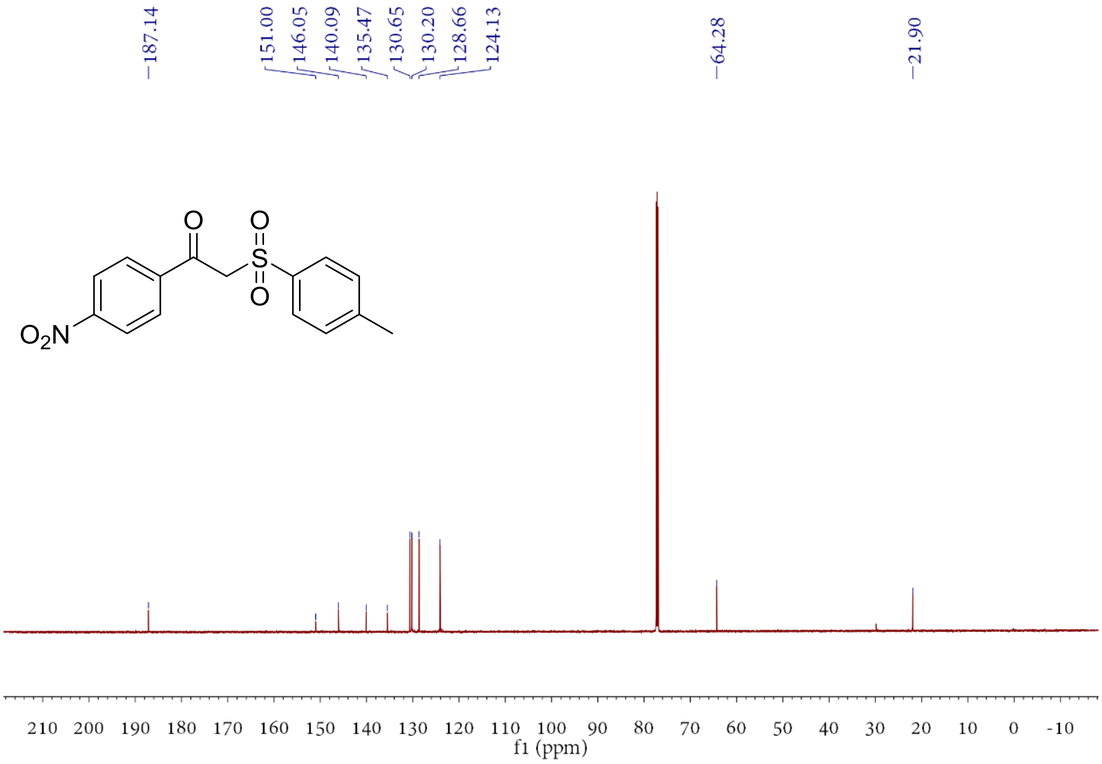

^{13}C NMR spectrum of compound **3h**

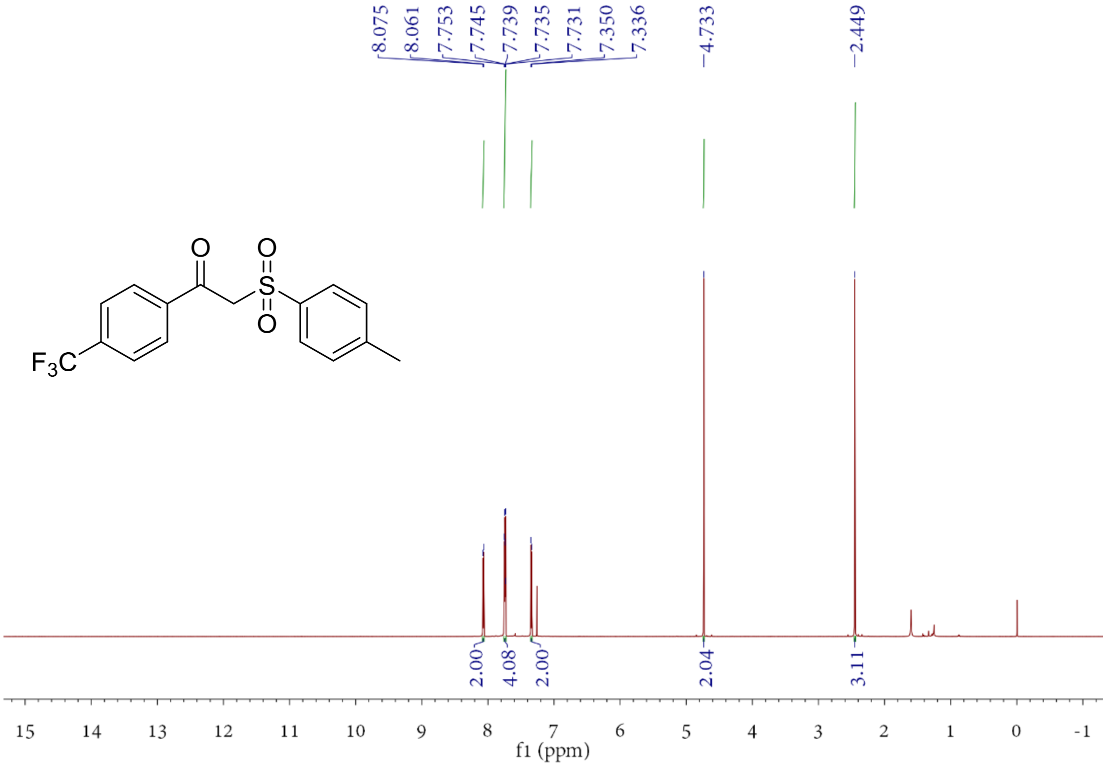

^{1}H NMR spectrum of compound **3i**

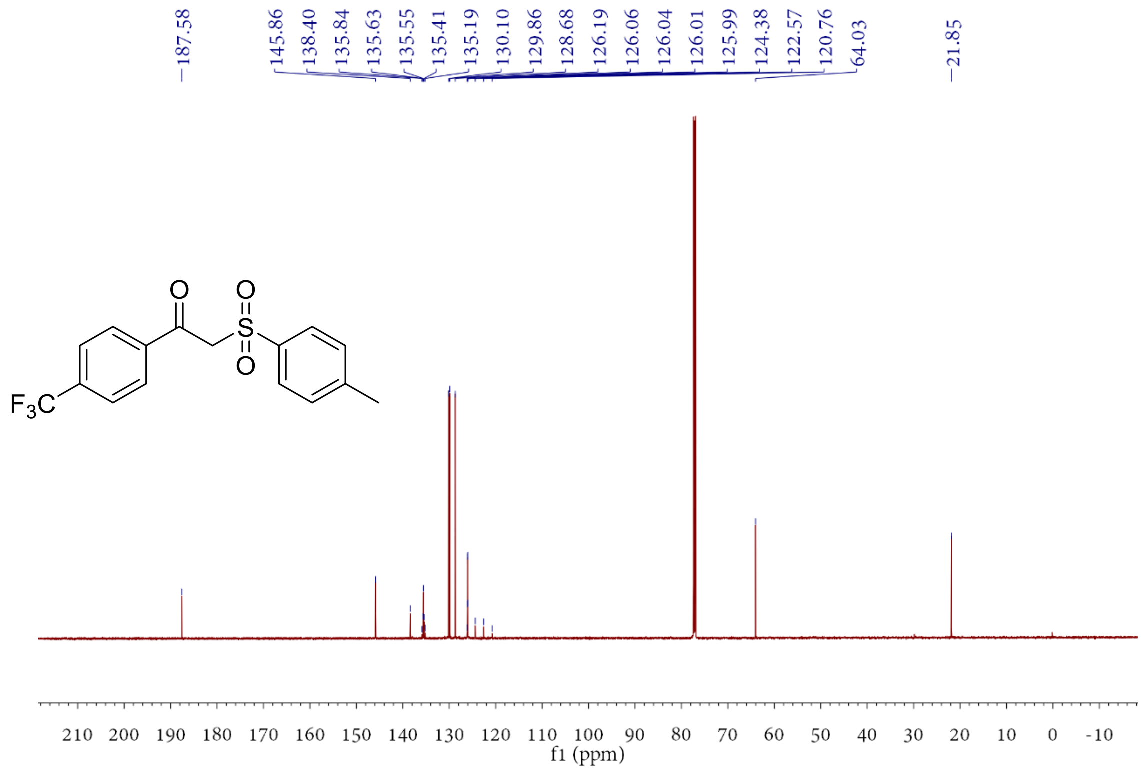

^{13}C NMR spectrum of compound **3i**

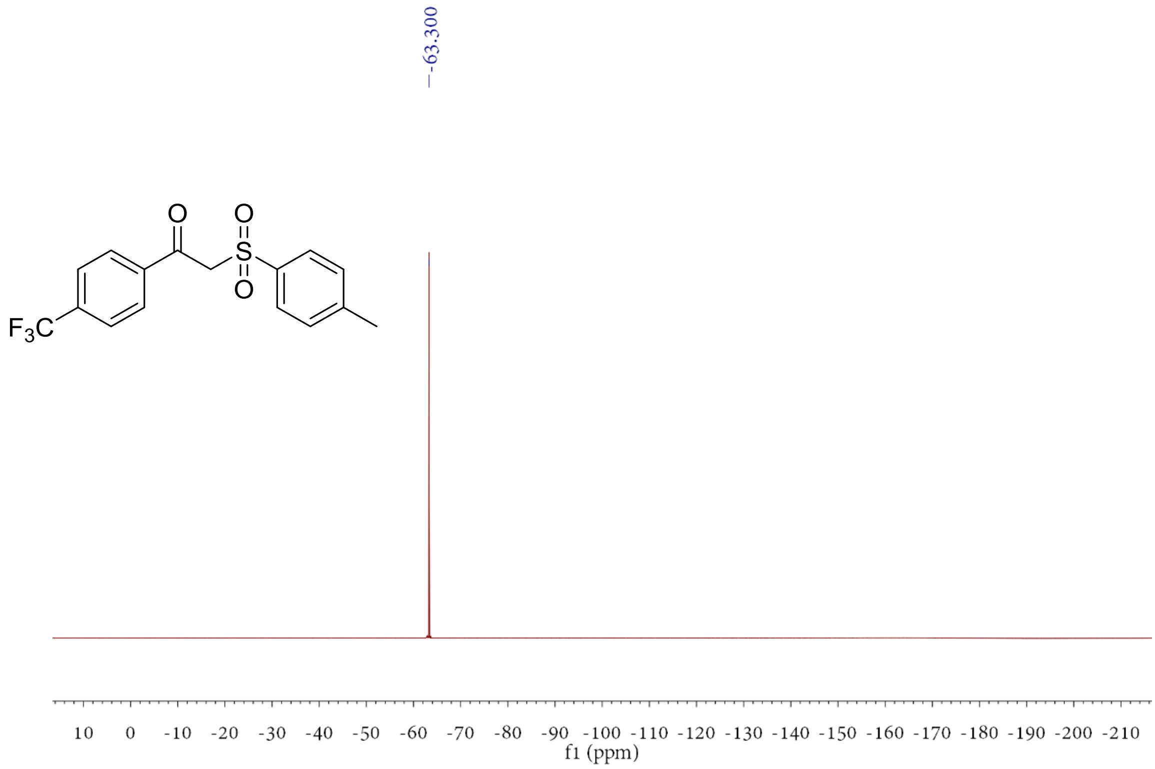

^{19}F NMR spectrum of compound **3i**

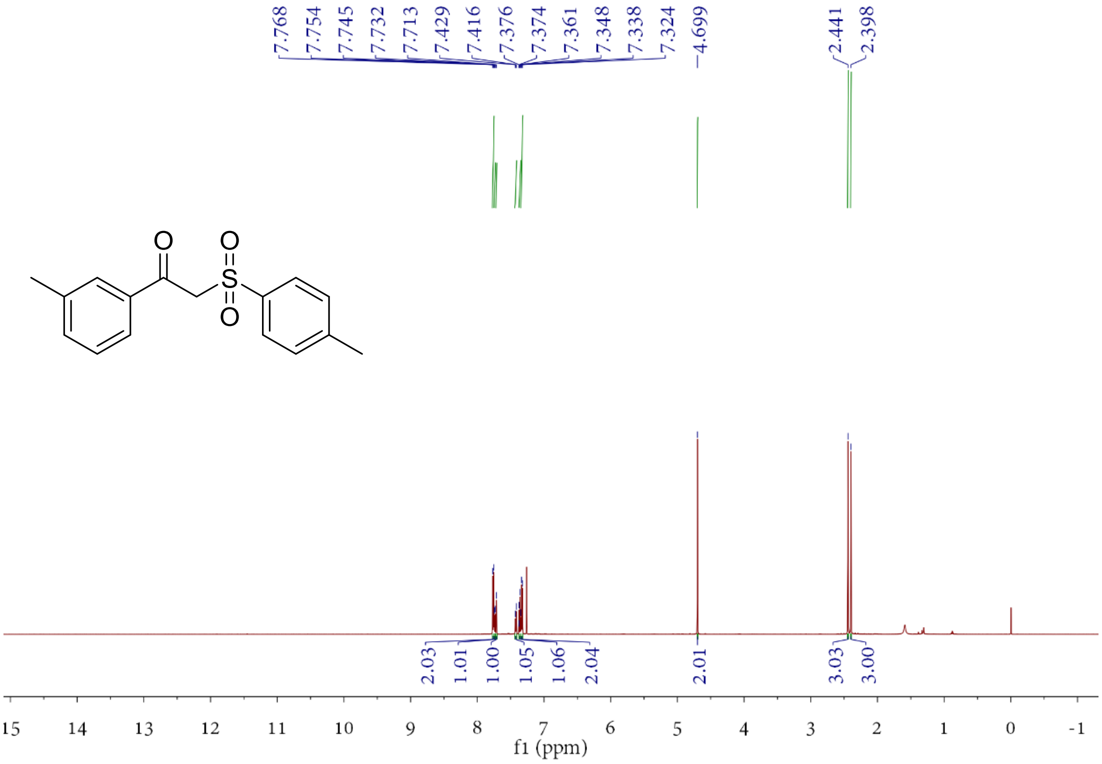

^{1}H NMR spectrum of compound **3j**

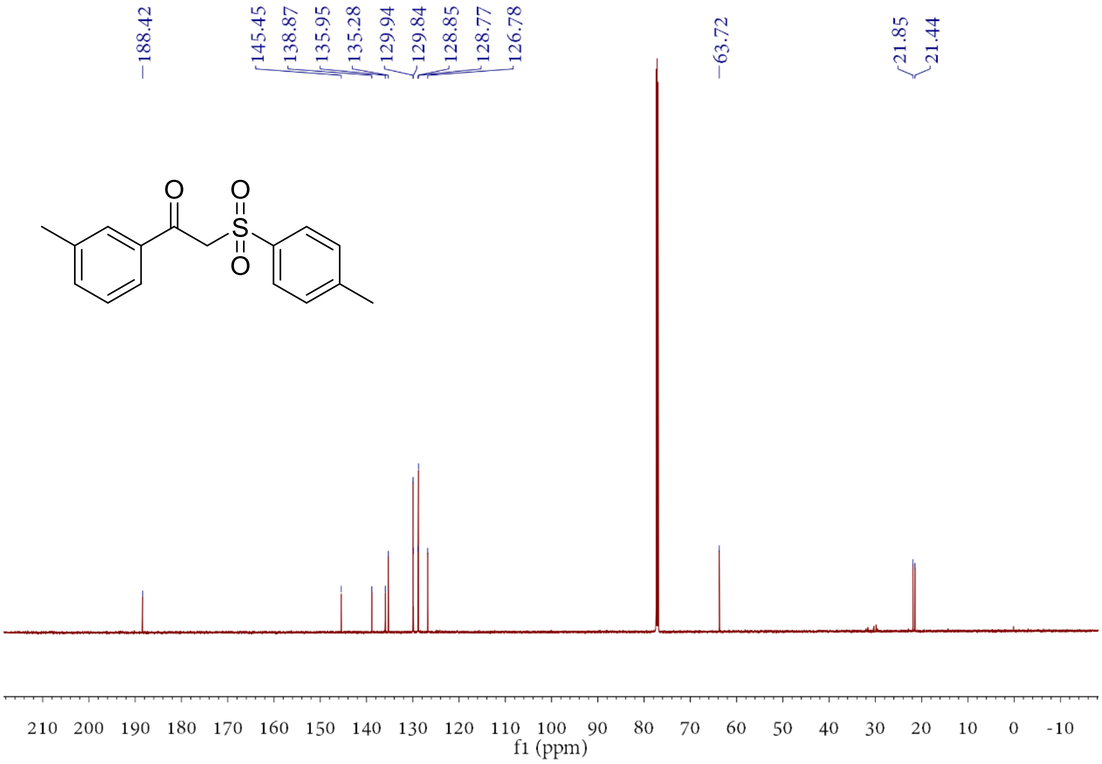

^{13}C NMR spectrum of compound **3j**

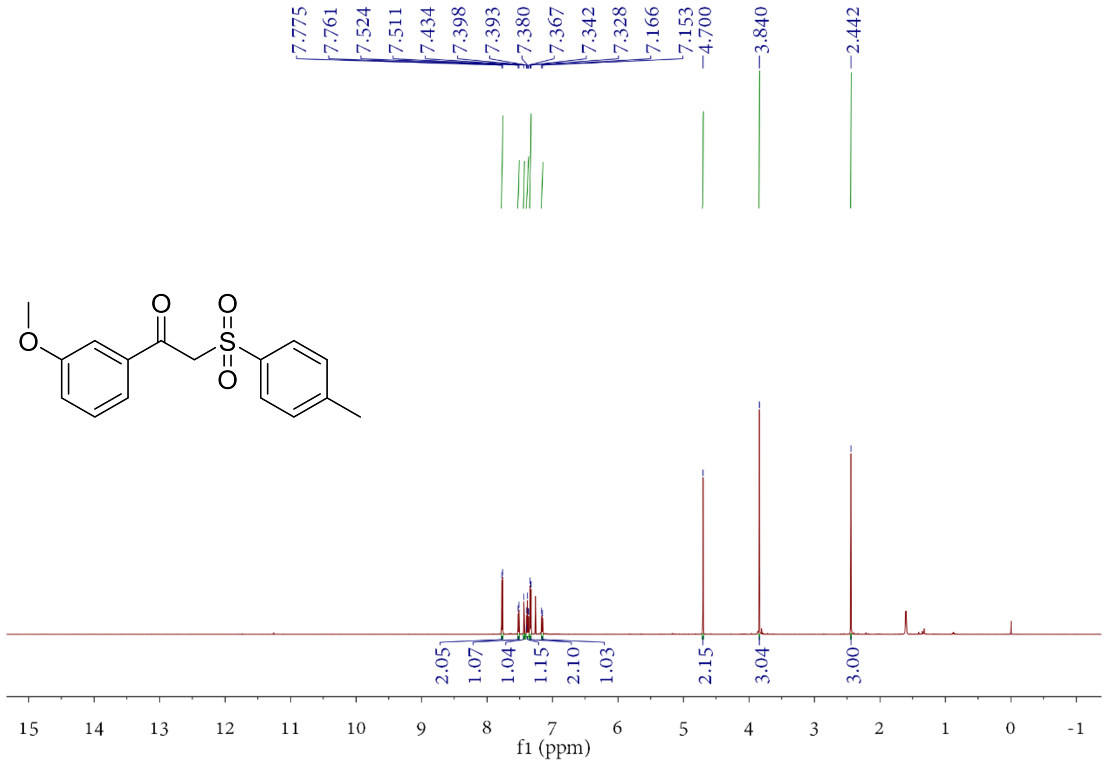

^{1}H NMR spectrum of compound **3k**

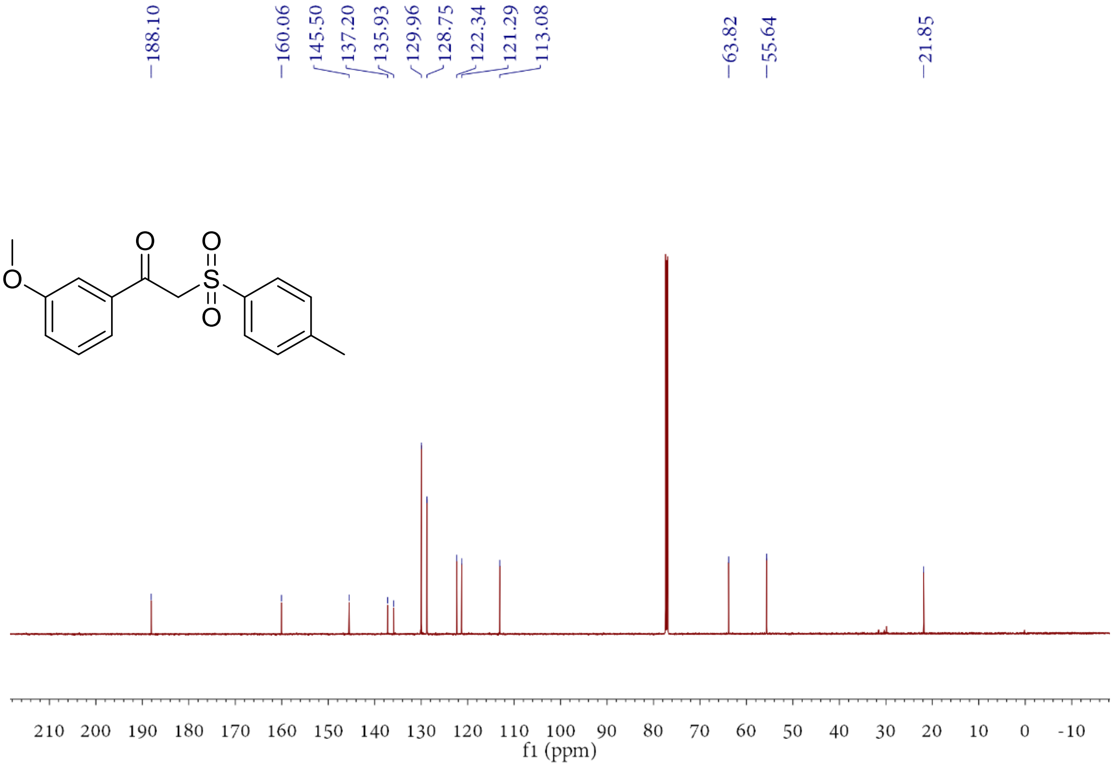

^{13}C NMR spectrum of compound **3k**

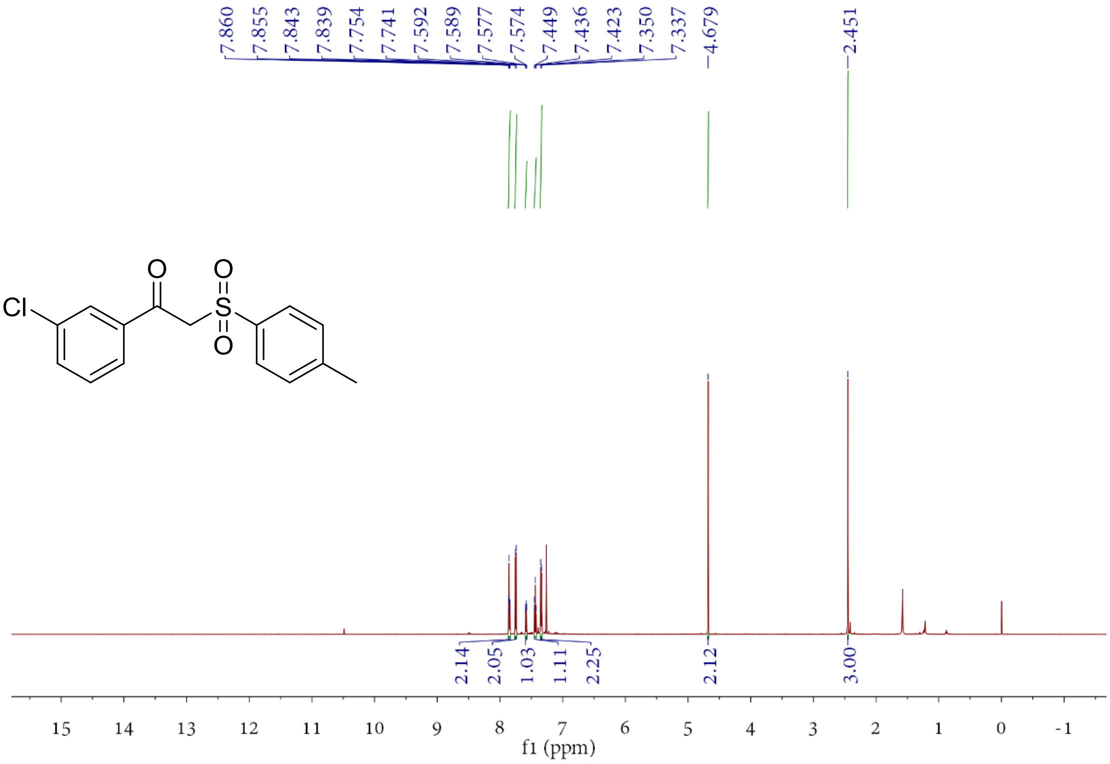

^{1}H NMR spectrum of compound **3l**

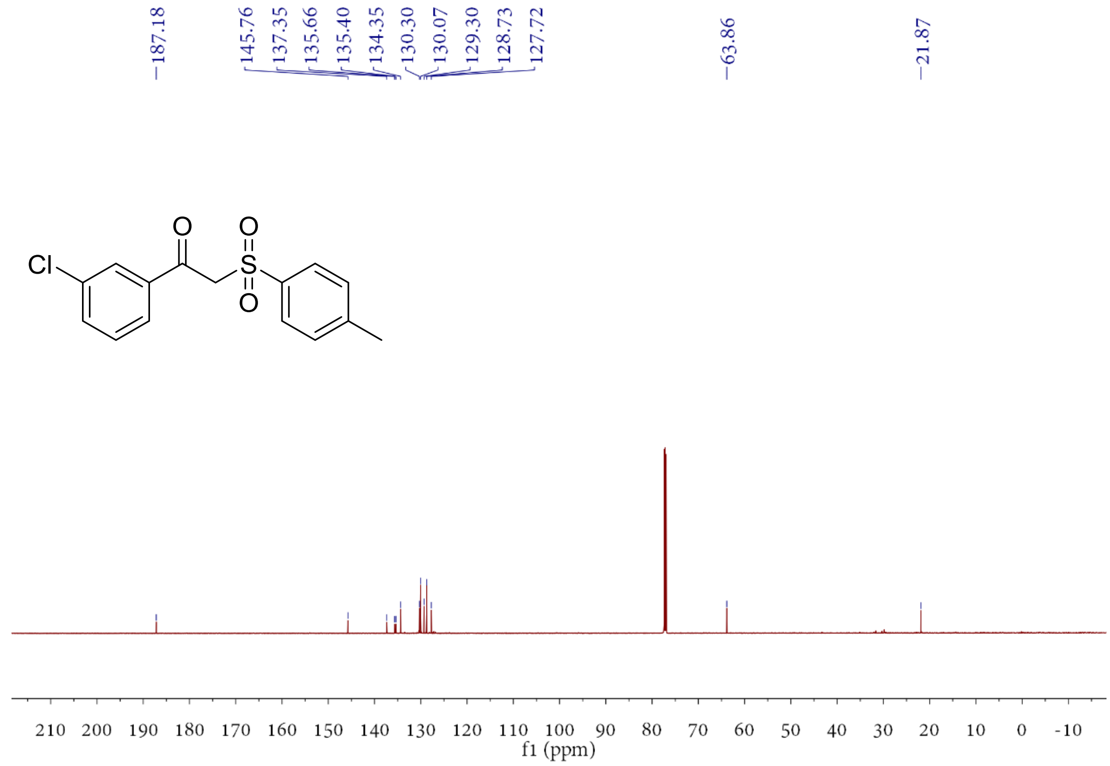

^{13}C NMR spectrum of compound **3l**

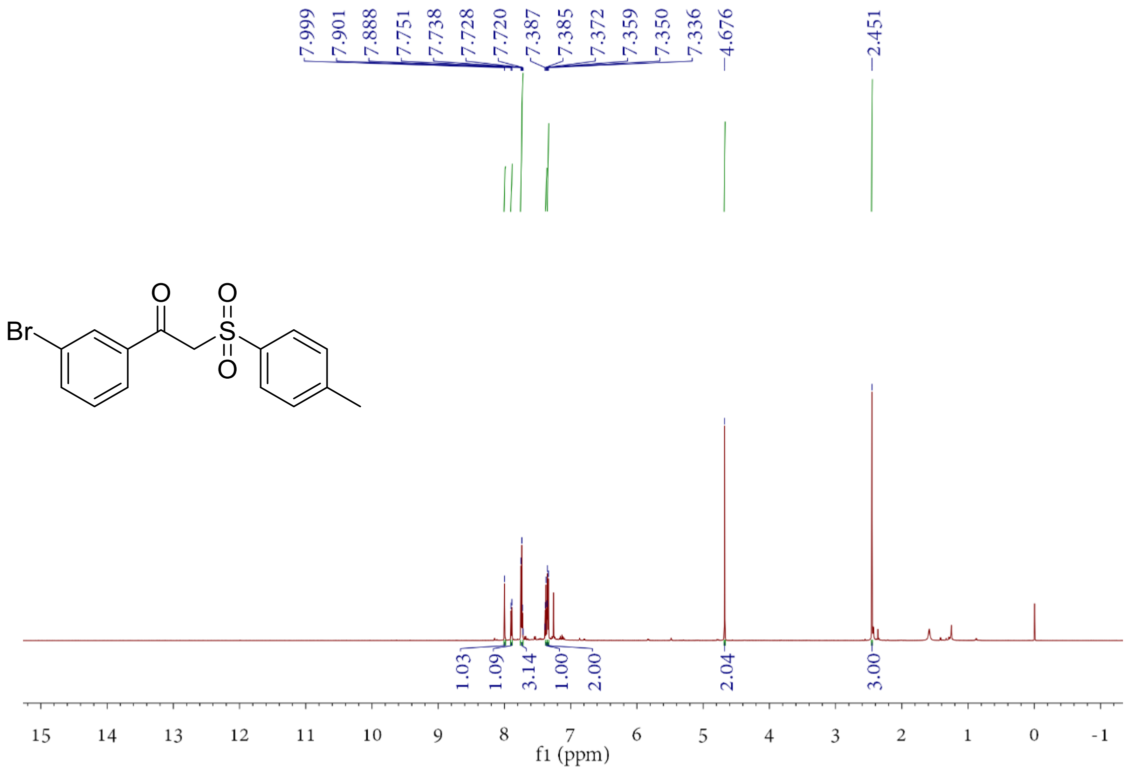

^{1}H NMR spectrum of compound **3m**

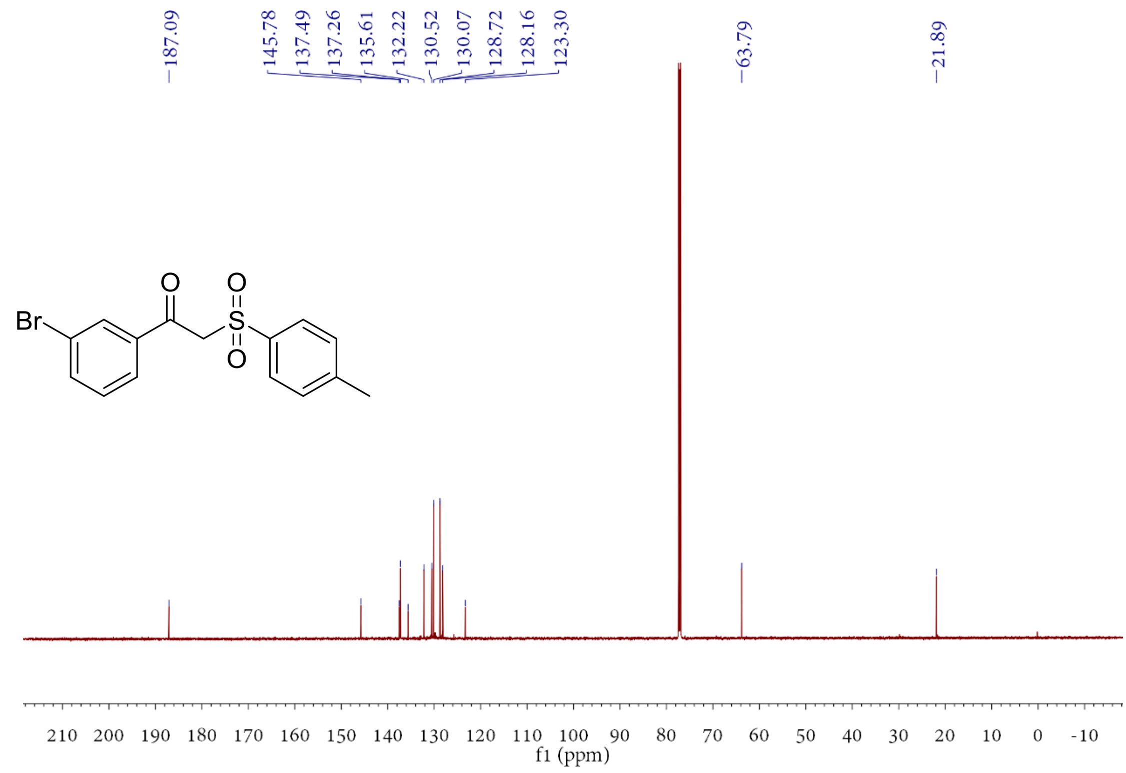

^{13}C NMR spectrum of compound **3m**

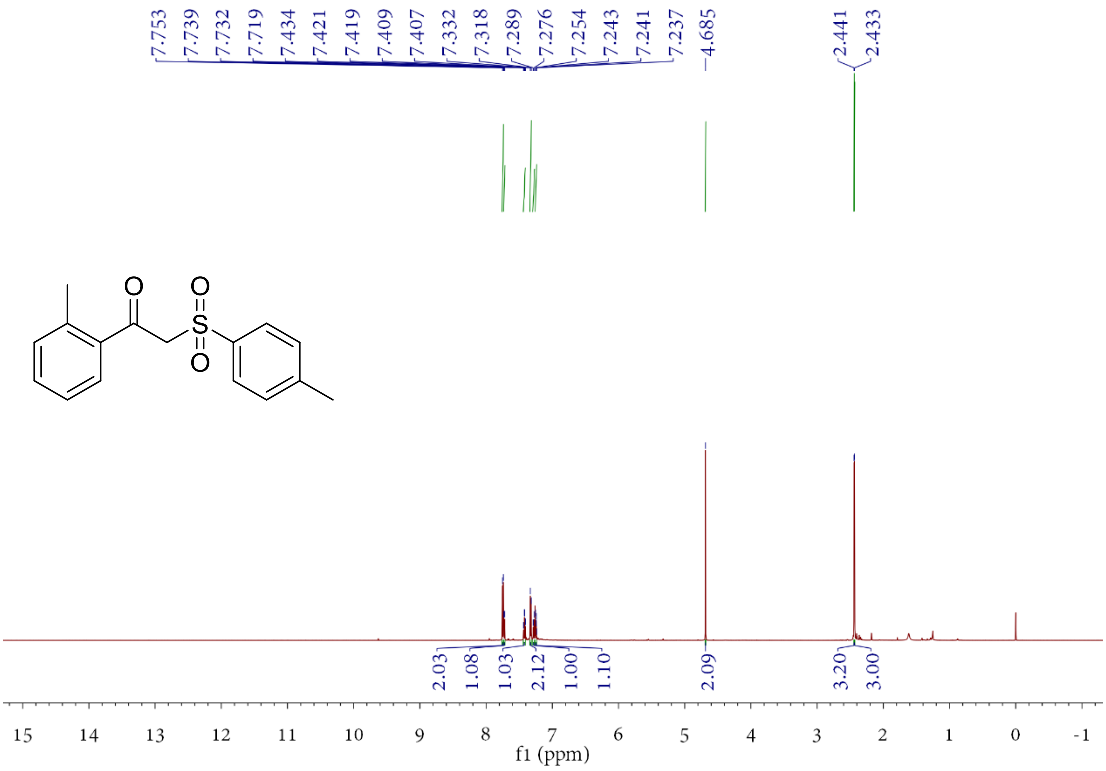

^{1}H NMR spectrum of compound **3n**

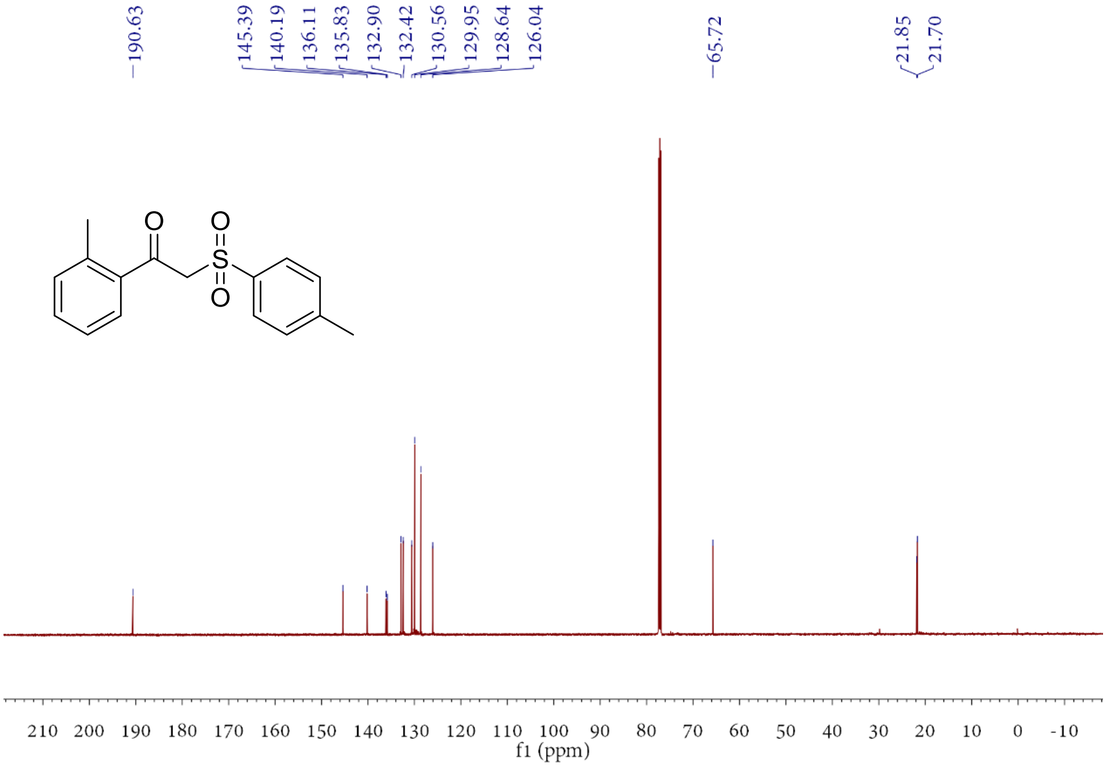

^{13}C NMR spectrum of compound **3n**

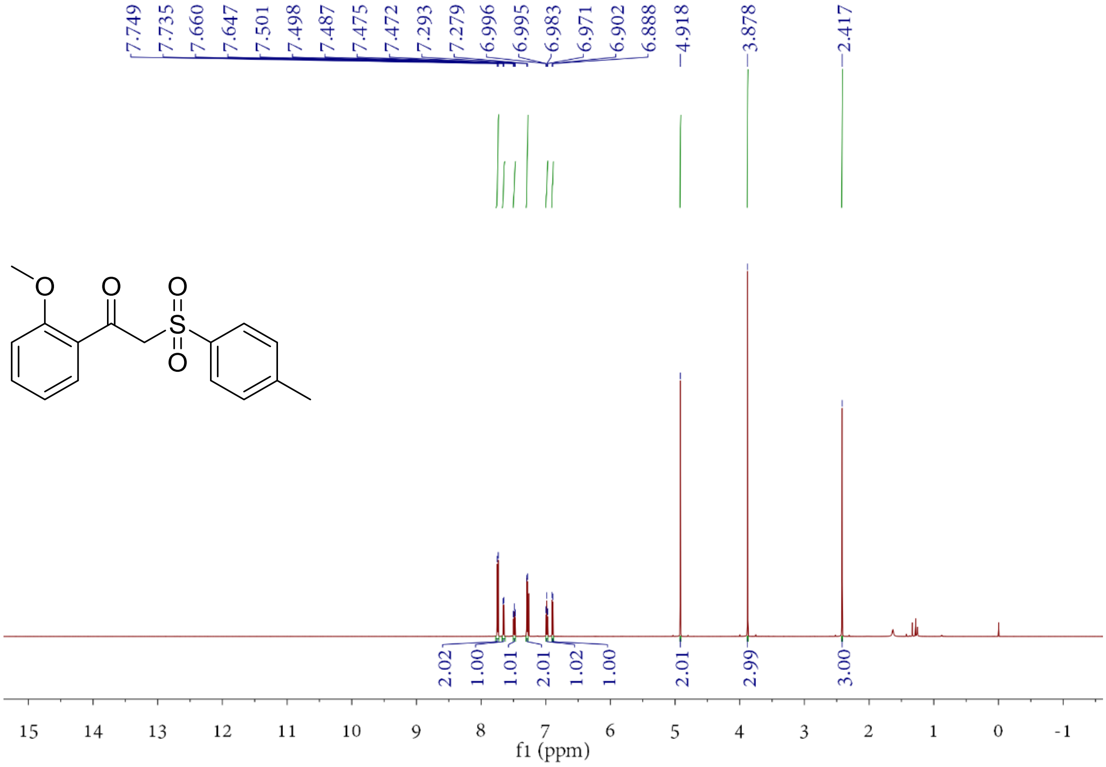

^{1}H NMR spectrum of compound **3o**

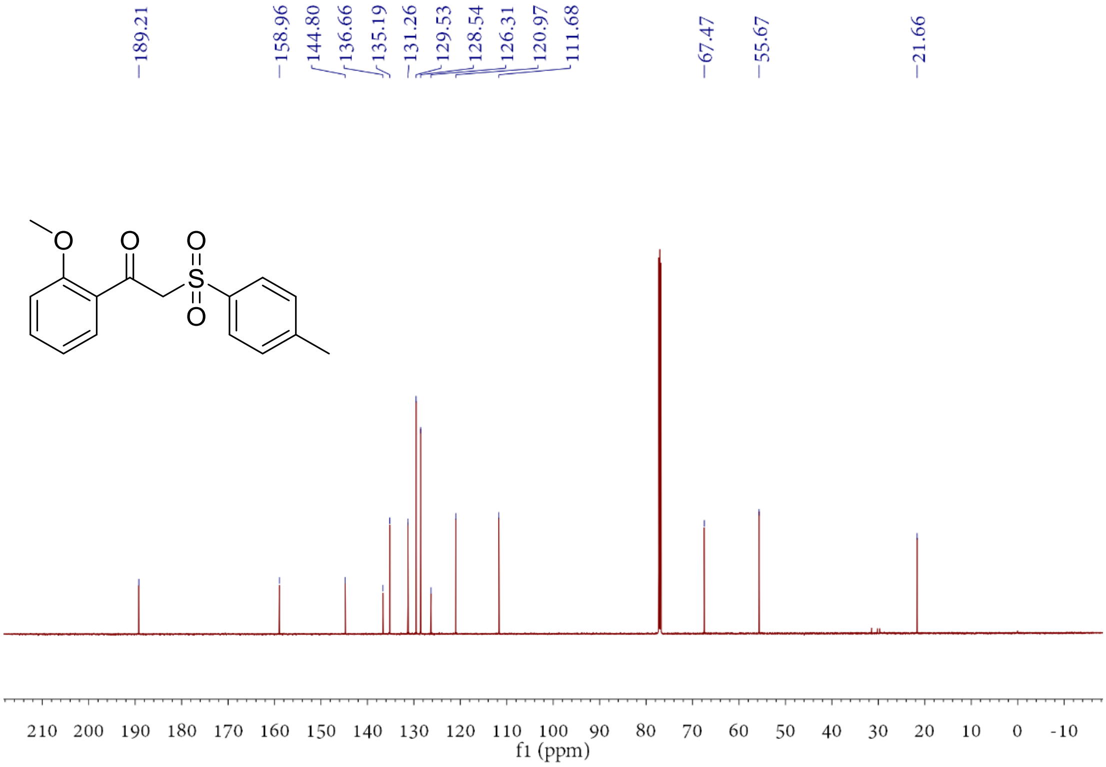

^{13}C NMR spectrum of compound **3o**

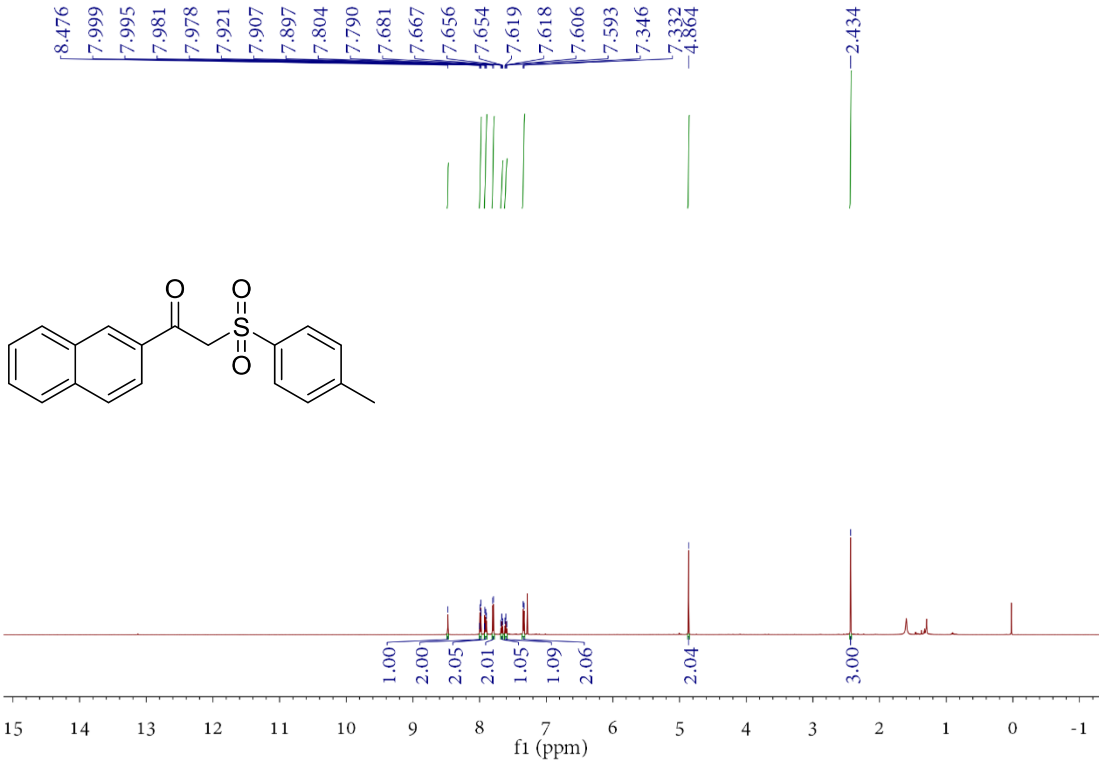

^{1}H NMR spectrum of compound **3p**

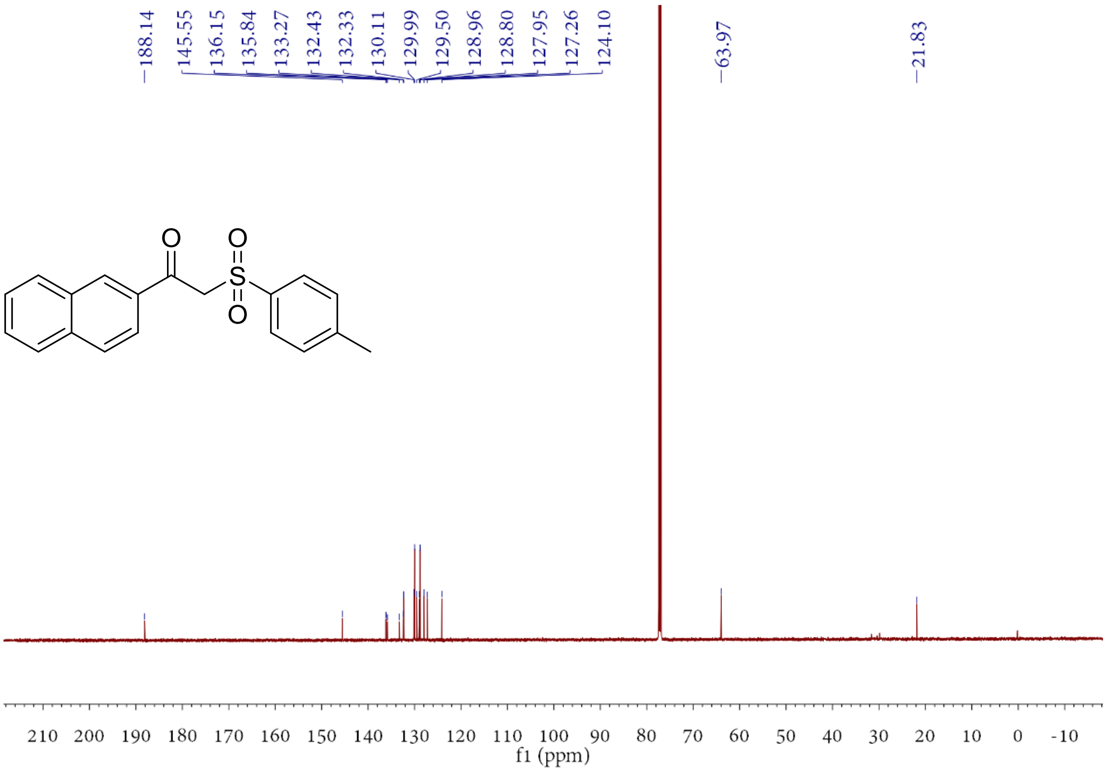

^{13}C NMR spectrum of compound **3p**

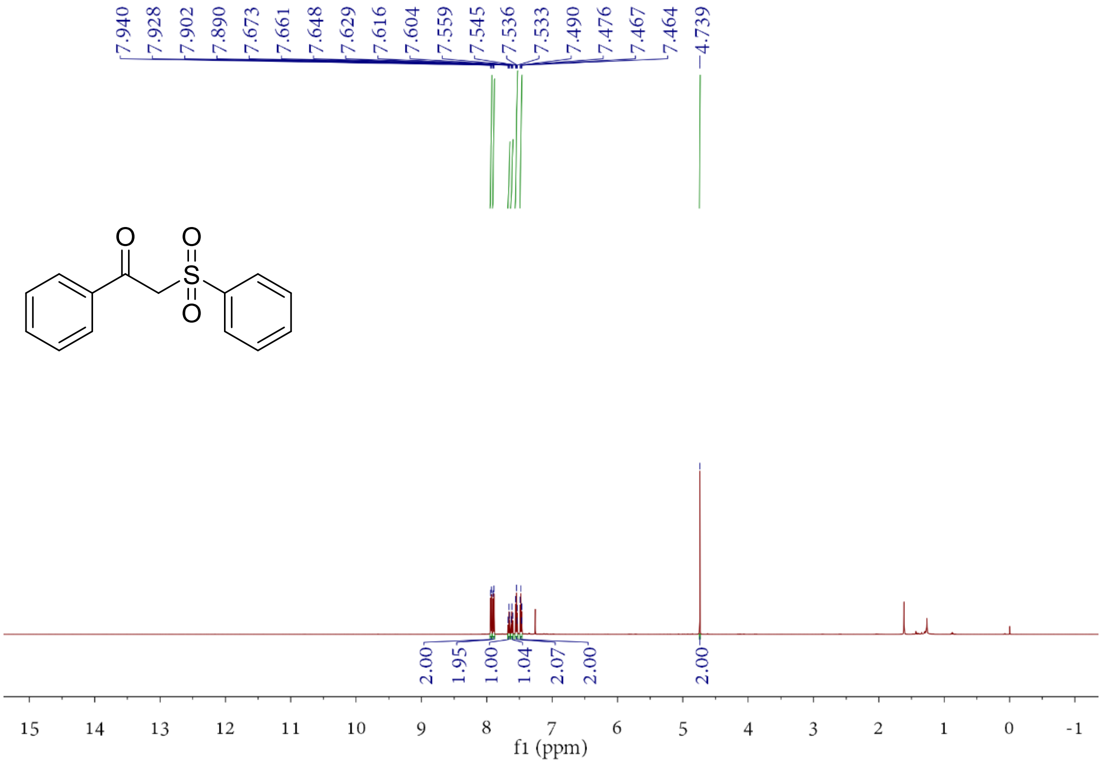

^{1}H NMR spectrum of compound **3q**

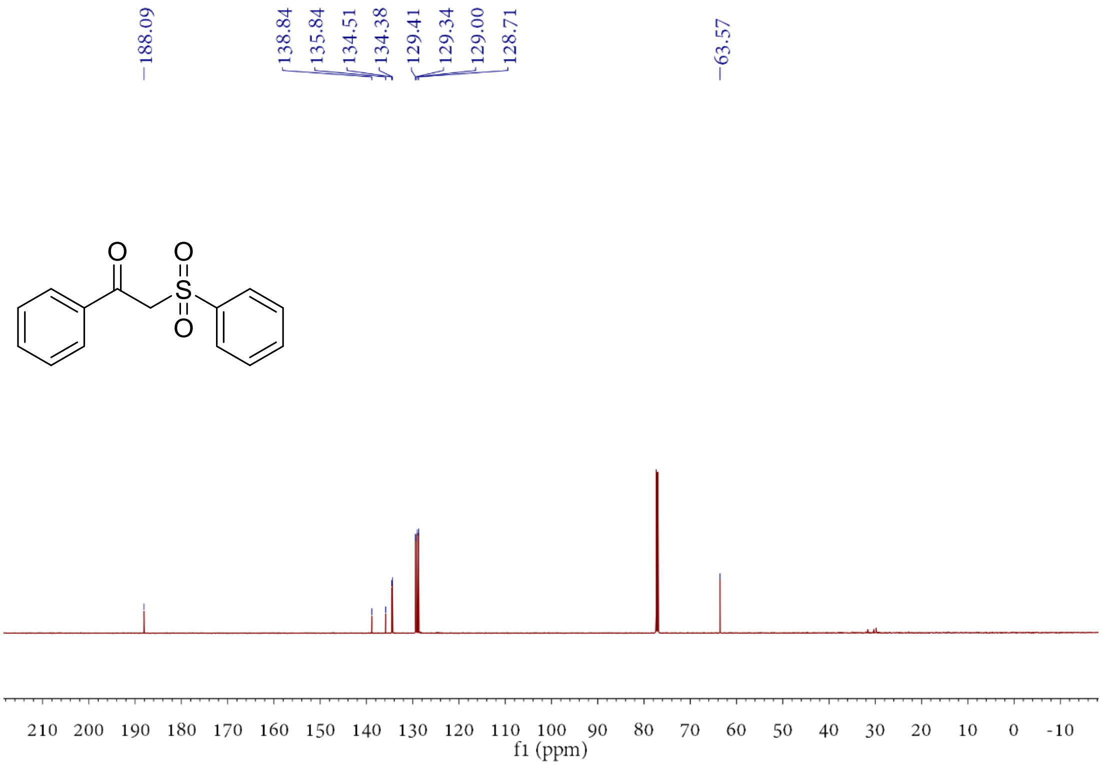

^{13}C NMR spectrum of compound **3q**

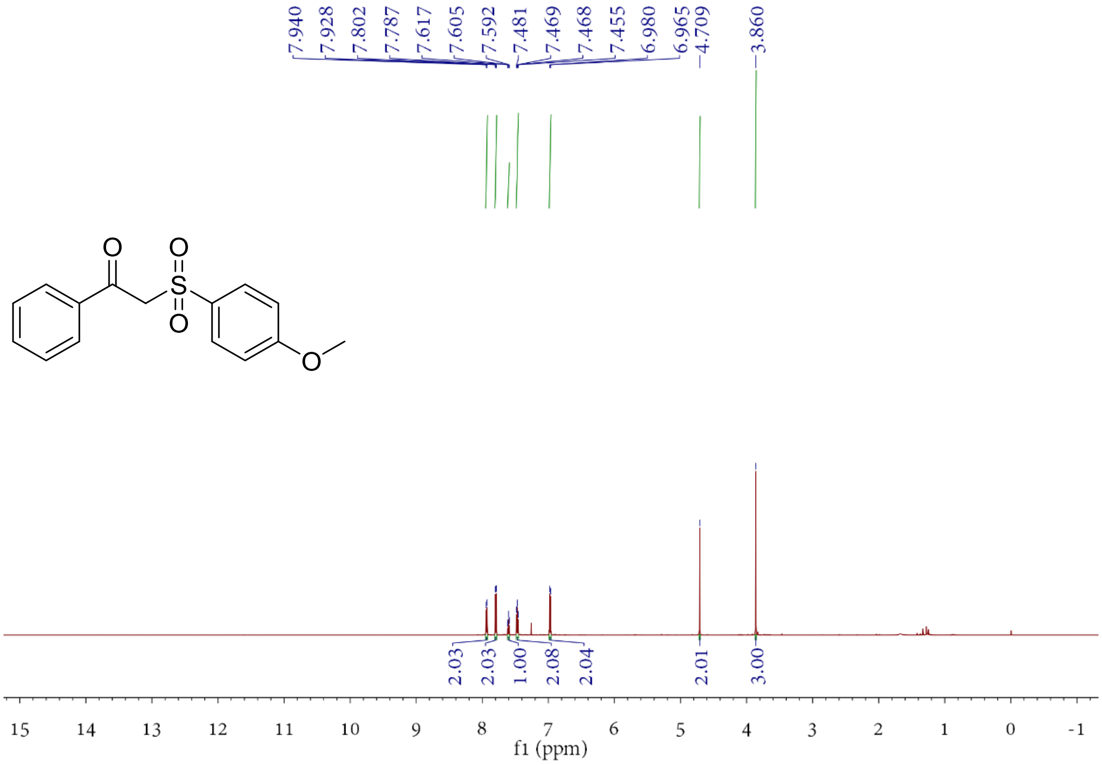

¹H NMR spectrum of compound **3r**

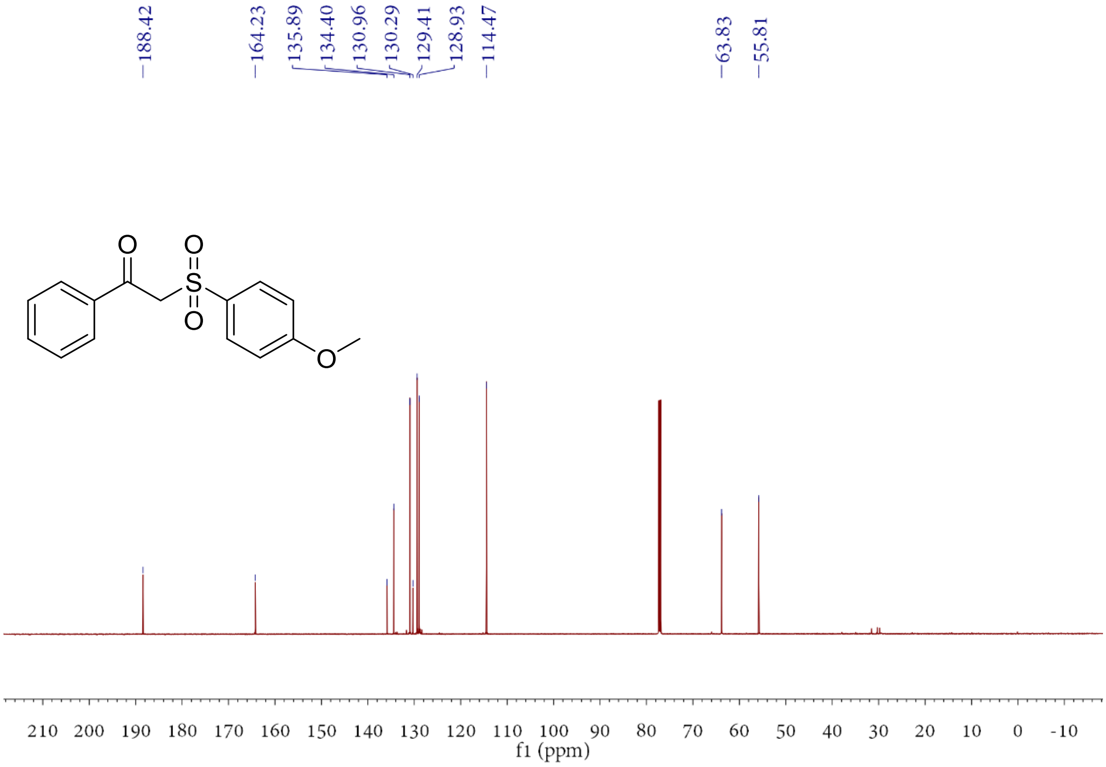

¹³C NMR spectrum of compound **3r**

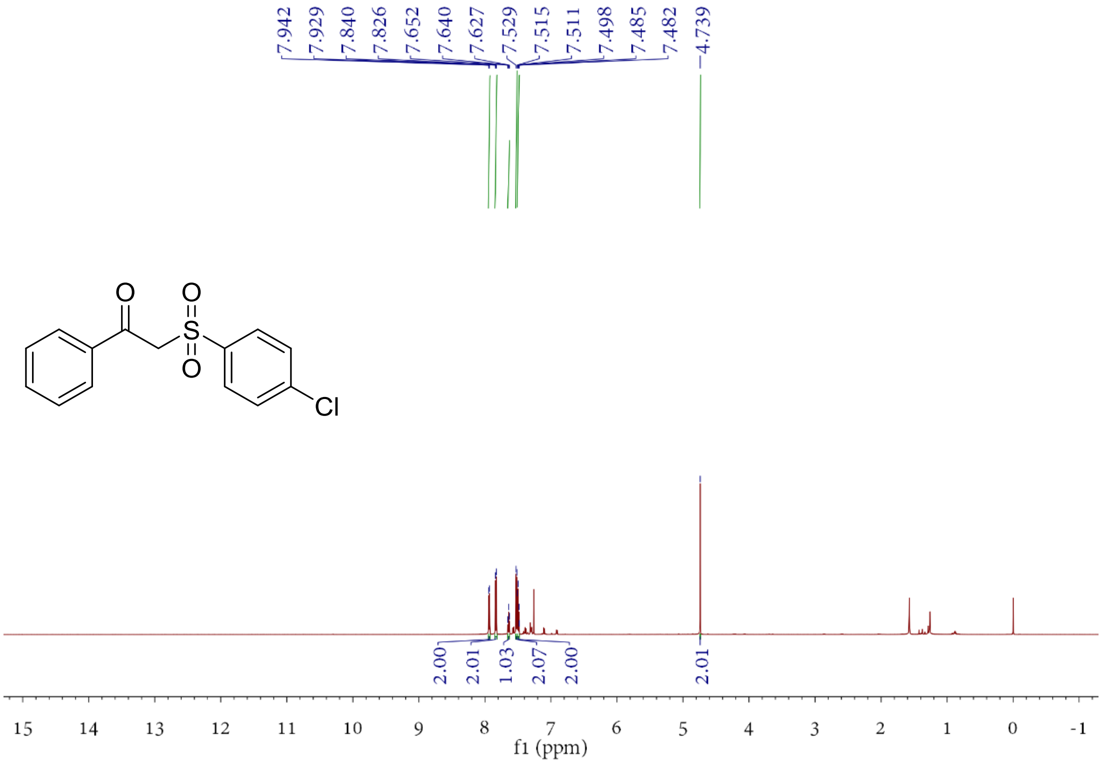

^{1}H NMR spectrum of compound **3s**

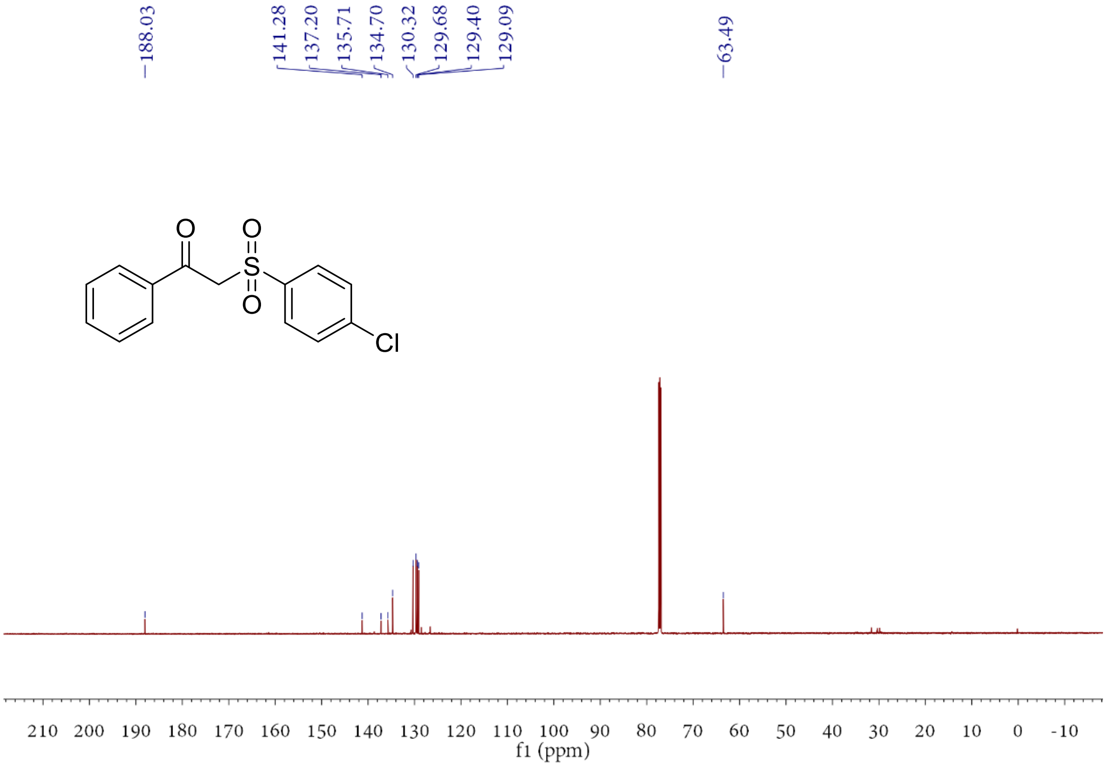

^{13}C NMR spectrum of compound **3s**

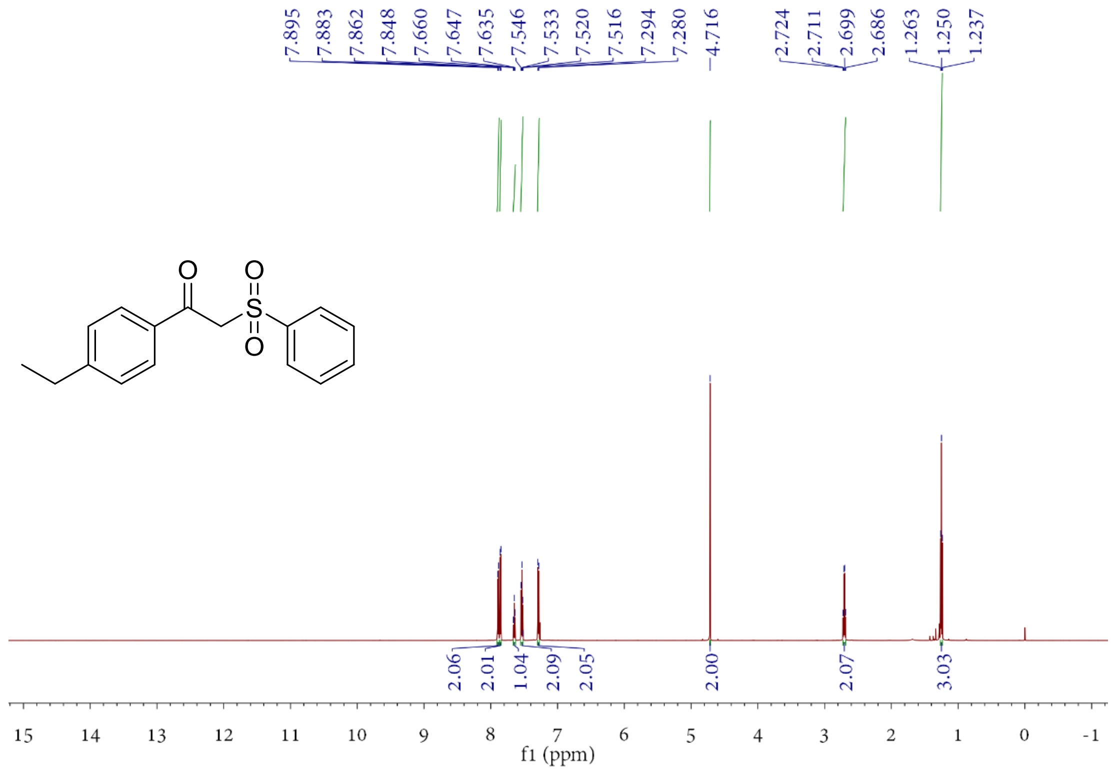

^{1}H NMR spectrum of compound **3t**

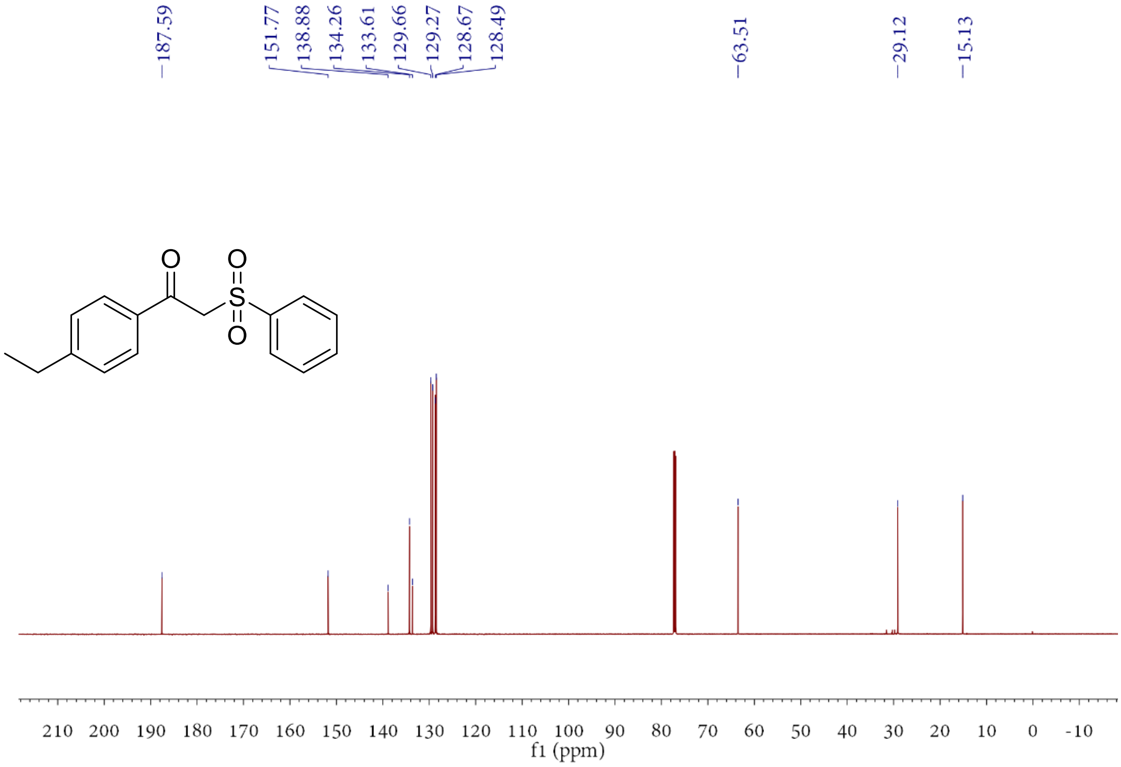

^{13}C NMR spectrum of compound **3t**

References

[1] B. N. Du.; P. Qian.; Y. Wang.; H. B. Mei.; J. L. Han.; Y. Pan. Cu-Catalyzed deoxygenative C2-sulfonylation reaction of quinoline *N*-Oxides with sodium sulfinate. *Org. Lett.,* **2016**, *18*, 4144-4147.
